# How does the crisis of the COVID-19 pandemic affect the interactions between the stock, oil, gold, currency, and cryptocurrency markets?

**DOI:** 10.3389/fpubh.2022.933264

**Published:** 2022-11-30

**Authors:** Jung-Bin Su, Yu-Sheng Kao

**Affiliations:** ^1^School of Finance and Accounting, Fuzhou University of International Studies and Trade, Fuzhou, China; ^2^College of Finance, Hubei University of Economics, Wuhan, China

**Keywords:** bivariate GARCH model, COVID-19 pandemic, volatility spillover, asset allocation, hedged strategy, hedge cost, risk reduction

## Abstract

This study examines how the COVID-19 pandemic crisis affects the interactions between the stock, oil, gold, currency, and cryptocurrency markets. The impacts of the COVID-19 pandemic crisis on the optimal asset allocation and optimal hedged strategy are also discussed. Empirical results show that the volatility spillover significantly exists in most of the ten paired markets whereas the return spillover and correlation are significant only for the few paired markets. Moreover, the impact of the COVID-19 pandemic on the return spillover is the greatest followed by the correlation whereas the volatility spillover is not affected by the COVID-19 pandemic. Furthermore, the Quantitative easing (QE) implemented after the COVID-19 pandemic crisis increases the risk-adjusted return for each asset and minimum variance portfolio (MVP) and raises the correlation between two assets. In addition, most of the pairs of assets are not suitable to hedge each other except for a few pairs of assets. Regarding these few pairs of assets, the optimal hedge asset with the fewer hedge cost is accompanied by less risk reduction and vice versa. Finally, the investors should choose the euro to construct a portfolio to achieve risk diversification and to hedge gold or WTI to get the risk reduction. The above findings can help investors and fund managers make a useful investment strategy, optimal asset allocation, and effective hedged strategy. For example, the investors can use the volatility of one market to predict the volatility of another market and they can take a long position during the post-COVID-19 period but they should withdraw capital from the market when the QE tapering is executed.

**JEL classification:**

C52; C53; G15.

## Introduction

Owing to the internet popularly used worldwide, information can be rapidly transmitted into any region. This leads the price of the financial market immediately to fluctuate as an extreme event occurs. For instance, most assets especially in the oil and stock markets immediately decreased in price level owing to information about the COVID-19 pandemic being spread out.^1^ Moreover, capital can freely flow in and out of any market and any country around the world due to globalization and liberalization. For example, the capital may be released by the quantitative easing (QE) policy mainly by the US during the post-COVID-19 period, and it is used to rescue the decreasing price level of assets owing to the COVID-19 pandemic. In addition, the assets in the stock, oil, gold, and currency markets play important roles during the process of the enterprise's operation.^2^ More importantly, cryptocurrency is a decentralized digital currency and has the following advantages the traditional currency never owns: easy transactions, incredible security, short settlement times and low fees, exponential industry growth, outsized returns, more private transactions, portfolio diversification, inflation hedge, cross-border payments, a more inclusive financial system, transactional freedom and 24 trading hours.^3^ Hence, cryptocurrency is a new and crucial asset. Then, we should consider it in this study. Thus, the Dow Jones index, WTI crude oil, gold, Chinese yuan (CNY), Euro, Bitcoin, Ethereum, and Litecoin in the stock, oil, gold, currency, and cryptocurrency markets of this study may interact with each other owing to the following two channels such as the information flow and capital flow, and one enterprise operation process.^4^ This indicates that the price return and its volatility for the above eight assets will spill over each other or return and volatility spillovers may exist between the five markets in this study. However, the COVID-19 pandemic, an extreme event, significantly affects the trend of the price level in the stock and oil markets when the information about the COVID-19 pandemic is spread out. The above phenomena motivate the issue of this study, how about the interactions between the stock, oil, gold, currency, and cryptocurrency markets, and how the crisis of the COVID-19 pandemic affects them.^5^ The obtained results can provide the investors to make a useful investment strategy, optimal asset allocation, and effective hedged strategy.

Subsequently, this study utilizes a diagonal bivariate BEKK-GARCH model with two time-dummy variables to explore the variation of correlation and return and volatility spillovers on the 23 pairs of assets or 10 paired markets for the pre- and post-COVID-19 periods.^6^ The obtained results are used to explore how the COVID-19 pandemic crisis affects the interactions between the stock, oil, gold, currency, and cryptocurrency markets. In addition, regarding each pair of assets for two subperiods, the optimal asset allocation is investigated by examining the risk diversification of a portfolio with minimum risk whereas the optimal hedge strategy is also explored by examining hedged cost and risk reduction of a hedged portfolio with minimum risk.^7^ The got results are utilized to discuss the impact of the COVID-19 pandemic crisis on the optimal asset allocation and optimal hedged strategy. Empirical results show that, irrespectively of the pre- or post-COVID-19 period, the volatility spillover significantly exists in most of the ten paired markets whereas the return spillover and correlation are significant only for the few paired markets. Moreover, from the viewpoint of the significant case appearing or disappearing after the COVID-19 pandemic, the impact of the COVID-19 pandemic on the return spillover is the greatest followed by the correlation whereas the volatility spillover is not nearly affected by the COVID-19 pandemic. Furthermore, the QE implemented after the COVID-19 pandemic crisis increases the risk-adjusted return for each asset and minimum variance portfolio (MVP) and raises the correlation between the two assets. In addition, most of the pairs of assets are not suitable to hedge each other except for a few pairs of assets. Regarding these few pairs of assets, the optimal hedge asset with the fewer hedge cost is accompanied by less risk reduction and vice versa. Finally, in eight assets Euro owns the lowest return and the smallest risk whereas Ethereum bears the greatest return and the highest risk. Moreover, within eight assets Euro and Ethereum, respectively, take the most and least capital weight in constructing an optimal portfolio. In addition, when hedging WTI or gold, Euro can get the greatest risk reduction whereas Ethereum can spend the cheapest hedge cost. Based on the above findings, we propose some policy implications for the investors and fund managers to make an effective investment strategy, optimal asset allocation, and effective hedged strategy.

The remainders of this paper are organized as follows. Section Literature review reviews the past literature about the spillover issue and then highlights the contributions of this study. Section Methodology describes the empirical model utilized in this study, the diagonal bivariate BEKK-GARCH model with two time-dummy variables, and the theories of optimal asset allocation and optimal hedged strategy. Section Data and descriptive statistics states the basic statistical features of the return series for the eight assets in the stock, oil, gold, currency, and cryptocurrency markets during the overall period and its two subperiods, the pre- and post-COVID-19 subperiods. Section Empirical results analyzes the results of the empirical model and further explores the issues addressed in this study further. Finally, Section Conclusion concludes the main findings of Sections Data and descriptive statistics and Empirical results and proposes some policy implications for various market participants. In addition, the limitations of this study and the direction of future research are also discussed.

## Literature review

Various assets in the world may strengthen to interact with each other attributed to the increasing trend of globalization, liberalization, and the internet used popularly worldwide. Further, except for the above reasons, the assets in the stock, oil, gold, currency, and cryptocurrency markets may also interact with each other because of the process of the enterprise's operation. As can be seen in Table 1, most researchers in recent years have focused on the spillover issue on several assets in the same market, such as the stock market (1–3), currency market (4), and cryptocurrency market (21). Table 1 lists the literature related to return and volatility spillovers within the recent 10 years. Notably, Table 1B only lists the literature related to the spillover issues about the impact of the COVID-19 pandemic. Some literature in Table 1 investigated the spillover issues for two different markets or one paired market to study the interaction between two markets such as the stock and bond markets (5), stock and oil markets (6, 7, 9, 10), stock and currency markets (12–16), stock and cryptocurrency markets (18), and currency and cryptocurrency markets (30). Seldom has literature investigated the spillover issues for more than one paired market to study the interaction between more than two markets. For example, they explored the spillover issues in three dissimilar markets such as the stock, oil, and currency markets (19) and the oil, gold, and cryptocurrency markets (22) or even in four markets such as the stock, oil, gold, and bond markets (20) and the stock, oil, gold, and cryptocurrency markets (23). Unlike the above literature, this study examines the spillover issues between eight assets in five different markets such as the stock, oil, gold, currency, and cryptocurrency markets. Or, we explore the spillover issues of the ten paired markets, which are the stock-oil, stock-gold, stock-currency, stock-cryptocurrency, oil-gold, oil-currency, oil-cryptocurrency, gold-currency, gold-cryptocurrency, and currency-cryptocurrency paired markets.^8^ To the best of my knowledge, five different markets or the ten paired markets explored in this study are the greatest numbers in the literature related to spillover issues. This is the study's first contribution to the literature because the types of markets examined in this study are more extensive than the literature about spillover issues before.

**Table 1:** Overview of the selected previous studies which analyze the return and volatility spillovers.

**Authors**	**Data and Period**	**Methods**	**Findings**
**A. The spillover literature with the various asset classes**			
Beirne et al. (1)	Data: the stock indices in 41 emerging market economies in Asia, Europe, Latin America, and the Middle East; Period: 1993/9–2008/3	Tri-variate VAR-BEKK-GARCH(1,1)-in-mean model	Spillovers from regional and global markets to local markets exist in the majority of EMEs. The nature of cross-market linkages varies across countries and regions
Allen et al. (2)	Data: stock indices in China, USA, Australia, Hong Kong, Japan, and Singapore; Period: period 1 (1991/8–1992/6); period 2 (1992/7–1996/12); period 3 (1997/1–2006/12); period 4 (2007/1–2010/11)	GARCH, VARMA–GARCH, VARMA–AGARCH models	Volatility spillovers exist across these markets in the pre-GFC periods, but there is little evidence of spillover effects from China to related markets during the GFC
Gilenko and Fedorova (3)	Data: stock indices in BRIC, SP500, DAX, Nikkei, and EMI; Period: pre-crisis period (2003/4–2007/12); crisis period (2008/1–2009/3); recovery (post-crisis) period (2009/3–2012/7)	4-dimensional BEKK-GARCH-in-mean model	There were some lagged mean-to-mean spillovers between the BRIC stock markets. Volatility-to-volatility spillovers between these stock markets are largely present
Kitamura (4)	Data: euro, the pound, and the Swiss franc; Period: 2008/7-2009/7	Varying-correlation model of multivariate GARCH	Return volatility in the euro spills into the pound and the Swiss franc, and these markets are highly integrated with the euro
Dean et al. (5)	Data: Australian equity and government bond; Period:1992/1–2006/11	Bivariate DCC-GARCH model and BEKK-GARCH model	Negative bond market returns spillover into lower stock market returns. Bond market volatility spills over into the equity market but the reverse is not true
Sadorsky (6)	Data: Oil (WTI), Stock (WilderHill Clean Energy Index, ECO; and the NYSE Arca Technology Index, PSE); Period: 2001/1–2010/12	Multivariate GARCH models (BEKK, diagonal, constant conditional correlation, and dynamic conditional correlation)	The DCC model also presents evidence of evidence of a statistically significant short-term persistence volatility spillover from oil to stock (ECO)
Smales (7)	Data: Oil (WTI), Stock (S&P500), and geopolitical risk (GPR) index; Period: 1986/1–2018/5	Multivariate GARCH models (BEKK, diagonal, constant conditional correlation, and dynamic conditional correlation)	This DCC model shows short- and long-term volatility persistence for oil and stock prices, together with spillover effects that run from oil to stock returns
Yousaf and Hassan (9)	Data: Stock (China, India, Korea, Indonesia, Pakistan, Malaysia, Philippines, Thailand, and Taiwan), Oil (Brent); Period: 2000/1–2018/6 including the US subprime crisis period and the Chinese stock market crash period	Ling and McAleer's (8) VAR-GARCH model	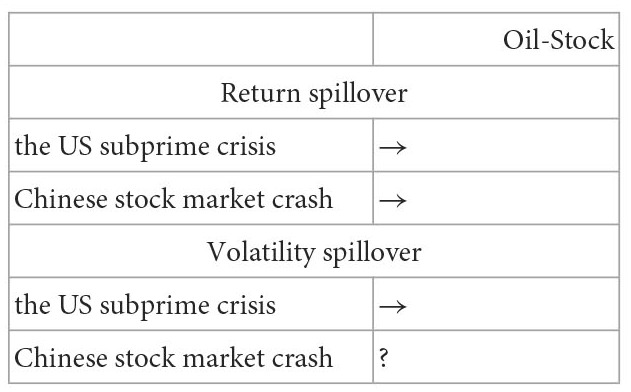
Cevik et al. (10)	Data: Oil (WTI and Brent), Stock [Borsa Istanbul 100 (BIST) index]; Period: 1990–2017	Univariate EGARCH model with Hong's (11) causality-in-mean test and causality-in-variance test	Return spillover: Oil→Stock; Volatility spillover: doesn't exist
Yang and Doong (12)	Data: the stock indices and exchange rate in the G-7 countries; Period: 1979/5–1999/1	Bivariate VAR with CCC-EGARCH-X model	Return spillover: Stock→FX; Volatility spillover: Stock→FX
Kumar (13)	Data: the stock indices and exchange rate in India, Brazil, and South Africa; Period: 2000/1–2011/1	VAR framework with the spillover index of Diebold and Yilmaz and multivariate BEKK-GARCH model	Return spillover: Stock→FX; Volatility spillover: stock→FX
Su (14)	Data: the stock indices and exchange rate in UK, Switzerland, Japan, South Korea, Singapore, Taiwan, and India; Period: 2001–2012	Univariate AR(1)-EGARCH(1,1)-X model	Return spillover: FX→Stock; Volatility spillover: FX→Stock
Sui and Sun (15)	Data: the stock indices and exchange rate in BRICS; Period: 2005–2014	VAR, variance decomposition, and impulse response functions	Return spillover: FX→Stock
Erdogan et al. (16)	Data: the Islamic stock indices and exchange rates in India, Malaysia, and Turkey; Period: 2013–2019	The Granger causality test and the causality-in-variance test of Hafner and Herwartz (17)	Return spillover: Stock→FX; Volatility spillover: Stock→FX in Turkey
Uzonwanne (18)	Data: Stock (CAC40, DAX, FTSE, Nikkei, S&P500), Cryptocurrency (bitcoin); Period: 2013/3–2018/3	Multivariate VARMA-AGARCH model	Return spillover: doesn't exist; Volatility spillover: Stock→Cryptocurrency
Su (19)	Data: Stock (Dow Jones, Nasdaq, and S&P500), Oil (WTI, GasNyh, and Heating), FX (UDI); Period: 2003/10–2015/8	Bivariate VAR with BEKK-GJR-GARCH-MX-t model with a structural break	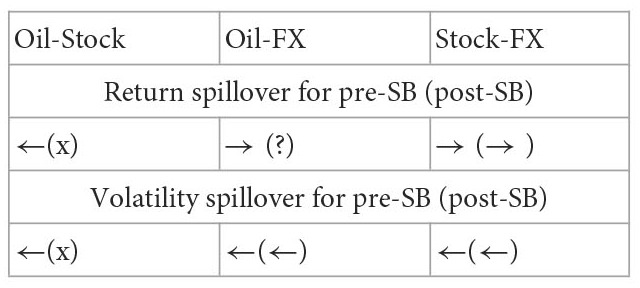
**B. The spillover of the covid-19 pandemic impact**			
Dutta et al. (20)	Data: Stock (S&P 500), Oil (WTI), gold, and Climate Bond; Period: 2017/3–2020/6	Bivariate VAR-ADCC-GARCH model	There is a bidirectional volatility linkage between climate bonds and the three indexes under study, whereas return linkages are marginal
Yousaf and Ali (21)	Data: Bitcoin, Ethereum, and Litecoin; Period: the pre-COVID-19 period (2019/1–2019/12) and the COVID-19 period (2020/1–2020/4)	VAR-DCC-GARCH model	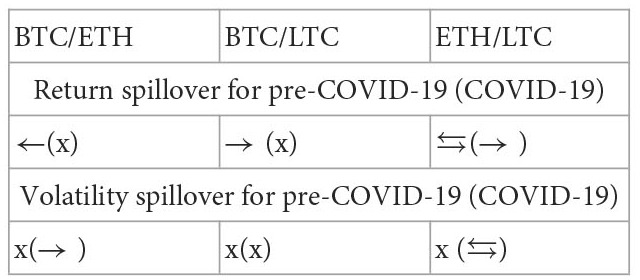
Yousaf et al. (22)	Data: oil, gold, and Bitcoin; Period: the pre-COVID-19 period (2019/5–2019/12; the COVID-19 period (2020/1–2020/5)	VAR-DCC-GARCH model	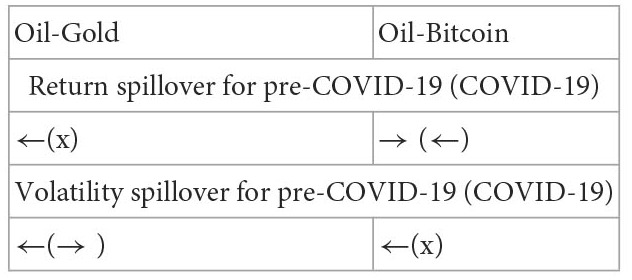
Arfaoui and Yousaf (23)	Data: S&P 500, WTI oil, Bitcoin, gold; Period: before COVID-19 (2015/1–2019/12) and during COVID-19 (2020/1–2021/8)	Multivariate VAR asymmetric BEKK GARCH model	Return spillover: oil market is the most affected market in the before COVID-19 period but gold is the major receiver in the COVID-19 period; Volatility spillover: oil market is very sensitive to gold and US stock markets, especially during the COVID-19 outbreak
Zaremba et al. (24)	Data: the term spread (TERM), the change in the number of COVID-19 infections, the relative rate of change in central bank total assets, broad Government Response Index, Containment and Health Index, and economic response Index; Period: 2020/1–2020/9	Panel regressions	First, the expansion of the disease significantly affects sovereign bond markets. Second, the growth of confirmed cases significantly widens the term spreads of government bonds. Third, an increase in the relative rate of change in the central bank balance sheet total assets exerts a negative effect on the term spread
Aharon et al. (25)	Data: the yield curve of G-7 countries and MCI (Media Coverage Index); Period: 2020/1–2021/8, covering the entire COVID-19 crisis	TVP-VAR methodology	The MCI and USA are the leading transmitters of spillover across all the yield curves in the G-7 countries. Moreover, Japan is a consistent receiver of risk from the G-7 countries
Gubareva et al. (26)	Data: the emerging market (EM) bond with the investment grade (IG) and high yield (HY); Period: 2020/1–2021/12	TVP-VAR methodology	The option-adjusted spreads (OAS) of the IG and HY financials have recovered to the pre-COVID levels by the end of the year 2020, while for the HY sovereigns and corporates the OAS remain twice as wide as before the pandemic
Umar et al. (27)	Data: five Non-Fungible Tokens (NFTs) (Art, Collectibles, Games, Metaverse, and Utilities) and Media Coverage Index (MCI); Period: 2020/1–2021/12	TVP-VAR methodology	Metaverse and Collectibles appear to be recipients of spillover for returns, whereas Art appears to be a net recipient of spillover for volatility. On the other hand, MCI appears to be a net transmitter for both return and volatility
Umar et al. (28)	Data: Seven high short interest indices (consumer, energy, financials, healthcare, industrials, real estate investment trusts, and technology), RavenPack Coronavirus MCI and Panic Index (PI); Period: 2020/2–2021/6	TVP-VAR methodology	The returns spillovers are more vigorous than the volatility spillover. Moreover, stocks of companies belonging to the energy and healthcare sectors are net recipients of returns and volatility spillover from the MCI
Umar et al. (29)	Data: spot price index of S&P GSCI gold, silver, platinum, and palladium, RavenPack COVID-19 induced global panic index (GPI); Period: 2020/1–2020/7	TVP-VAR methodology	First, the panic induced by COVID-19 is a shock transmitter to precious metals market. Second, we found silver to resist to these shocks while gold was a net receiver for almost all the period of analysis. Third, platinum and palladium on the other hand show a switching time varying patterns of connectedness to COVID-19 panic
Umar et al. (30)	Data: cryptocurrencies (Bitcoin, Ethereum, and Ripple), the fiat currencies (euro, GBP, and Chinese yuan), and the RavenPack Coronavirus MCI; Period: 2020/1–2020/12	TVP-VAR methodology	The media coverage index and the cryptocurrencies are the net transmitters of shocks while the fiat currencies are the net receivers of shocks
Umar et al. (31)	Data: the bond indices for the EM High-Yield, EM Investment Grade, and the US Treasuries, and RavenPack Coronavirus MCI; Period: 2020/1–2020/12	TVP-VAR methodology	Our results show a significant increase in the dynamic connectedness between media coverage, emerging market bonds, and US bonds, as well as between the respective volatilities, especially during the early phases of the COVID-19 pandemic, with the highest values observed in March 2020
Umar et al. (32)	Data: the volatility of the S&P GSCI spot commodity indices and the Ravenpack Coronavirus Panic Index (PI); Period: 2020/1–2020/7	Wavelet coherence methodology	There are intervals of low coherence across various time and frequency scales for these indices. The low coherence intervals show that diversification benefits
Ali et al. (33)	Data: the infectious disease-related equity market volatility (IDEMV) and bond indices (US, UK, Japan, Switzerland, Canada, Australia, Sweden, China, and Europe); Period: 2000/1–2021/2	Wavelet coherence methodology	The results show no significant co-movement between these bond indices and IDEMV, thus confirming that they serve as a hedge against IDEMV
Umar and Gubareva (34)	Data: the Bloomberg Galaxy Crypto Index (BGCI), fiat currencies (EUR, GBP, and RMB), and the Ravenpack Coronavirus PI; Period: 2020/1–2020/5	Wavelet coherence methodology	All the PI-currency pairs display similar patterns along the time and frequency scales in the respective heatmaps implying high coherence and interdependence around the apogee in the mid-March of the COVID-19 panic

1. TVP-VAR is the abbreviation of Time-varying parameter vector autoregression. 2. The symbol “Stock→FX” denotes there exists a spillover from the stock market to the exchange rate market (or currency market) so are the other symbols “Oil→Stock” and “Stock→Cryptocurrency.” 3. The symbols “→(←)” in column “oil-Bitcoin” at panel “Return spillover for pre-COVID-19 (COVID-19)” in Yousaf et al. (22) denotes that there exists a return spillover from oil to Bitcoin in the pre-COVID-19 period but from the bitcoin to oil in the COVID-19 period. At the same inference process, the symbols “←(x)” in column “oil-stock” at panel “Volatility spillover for pre-SB (post-SB)” in Su (19) denote there exists a volatility spillover from stock to oil in the pre-SB period but no spillover exists between the stock and oil markets in the post-SB period.

Moreover, there is a COVID-19 pandemic crisis during the study period. Then, this study utilizes a diagonal bivariate BEKK-GARCH model with two time-dummy variables to explore the variation of correlation and return and volatility spillovers on the ten paired markets for the pre- and post-COVID-19 periods.^9^ The variation of results for the two subperiods is utilized to examine the impact of the COVID-19 pandemic on the correlation and return and volatility spillovers between five markets. Recollections of the past literature on the spillover issues, most of them examined the spillover issues for the entire study period even if some extreme events happened during the study period such as the global financial crisis (4, 6, 7, 10, 14) and the COVID-19 pandemic (20). Then, their findings are unbelievable because the models don't consider the effect of extreme events and the results found just are the average phenomena of the spillover issues for that entire study period. Even if few works of literature divided the study period into several periods according to the dates of extreme events happening to explore the impact of this extreme event on the spillover. However, they individually estimate the model parameters for each subperiod (2, 3, 9, 21–23). For example, as reported in Table 1B, Yousaf and Ali (21), Yousaf et al. (22), and Arfaoui and Yousaf (23) divided the study period into the pre- and post-COVID-19 periods to explore the impact of COVID-19 pandemic on the spillover issues. Then, they executed the estimate of model parameters two times respectively for the pre- and post-COVID-19 periods. This indicates that for the estimate of parameters the above method need do several times but one time for our model. This is the study's second contribution to the literature. Because the bivariate BEKK-GARCH model with two time-dummy variables considers the effect of an extreme event and the parameter estimate of this model is more efficient than that of other methods with individually estimating the model parameters for each subperiod, which is partitioned by the date an extreme event occurring.^10^ Thus, our model is superior to most of the bivariate GARCH family models.

Furthermore, there are four types of approaches used in the literature on issues of spillover or interaction. The first approach used the bivariate GARCH family models to estimate the coefficients of cross-term in the mean equation and variance-covariance equation of these models to examine the return spillover and volatility spillover between two assets by using the significant situation of coefficients [see (1–7, 9, 12, 14, 18–23) and so on].^11^ The second approach used the time-varying parameter vector autoregression (TVP-VAR) methodology, which extends the connectedness work of Diebold and Yilmaz (38), to calculate the net total directional connectedness for each asset within a group of assets to determine whether this asset is a net transmitter or a net receiver on return or risk by using the value of net total directional connectedness being positive or negative [see (13, 25–31)]. However, this approach can't determine whether the spillover is significant or not and whether the spillover is positive or negative. Moreover, this approach can't find any result of the correlation between two assets because the VAR model on the mean return is used in this approach. The third approach used the *Q*_1_ and *Q*_2_ statistics, respectively, corresponding to Hong's (11) causality-in-mean test and causality-in-variance test to, respectively, inspect the return spillover and volatility spillover for a pair of assets. The two statistics are calculated based on the standardized residuals of two univariate models. Hence, this approach can determine whether the return (or volatility) spillover is significant or not *via* the test statistic *Q*_1_ (or *Q*_2_) [see (10, 16)].^12^ However, this approach can't determine whether the spillover is positive or negative. Moreover, it can't find any result of the correlation between two assets because the univariate GARCH models are used in this approach. The fourth approach utilizes wavelet coherence methodology to approximately measure the correlation between two assets on the time and frequency domain by observing the variation of color in the wavelet coherence figure. By observing the direction of the arrow, this approach can determine whether two assets have a positive relationship or negative correlation in the time and frequency domain [see (32–34)]. Hence, this approach can't give a statistically significant result of correlation owing to only illustrating a qualitative analysis of the correlation across the time and frequency by using the color images. Moreover, this approach can't find any results of return and volatility spillovers. Notably, this study uses both the network graphs and the results of a diagonal bivariate BEKK-GARCH model with two time-dummy variables to explore the impact of the COVID-19 pandemic on the correlation, return spillover, and volatility spillover for the ten paired markets. Even if our approach belongs to the first approach but as illustrated in the previous paragraph our model with two time-dummy variables can seize the impact of an extreme event and is more efficient for the model parameters estimated. Hence, our method is superior to the first approach. In addition, our approach is very efficient as compared with other methods such as Hong's (11) causality-in-mean and causality-in-variance tests, TVP-VAR methodology, and the wavelet coherence approach. This is the study's third contribution to the literature because for the correlation and both return and volatility spillovers the bivariate BEKK-GARCH model with two time-dummy variables can give statistically significant results and also can determine whether the results are positive or negative values. However, Hong's (11) causality-in-mean and causality-in-variance tests, TVP-VAR methodology, and the wavelet coherence approach can't completely give the above functions.^13^ As a result, our model is better than the other methods popularly used in the spillover literature in terms of determining whether the results are significant and whether they are a positive or negative value.

In addition, it is very difficult to explore the interaction issues between five markets by using the results of three types of interactions during two subperiods for 23 pairs of assets. In this study, we make some rules to simplify the results of 23 pairs of assets into the significant, slightly significant, or insignificant results of 10 paired markets. Thereafter, we follow Umar et al. (28) to construct the network graphs of return spillover, volatility spillover, and correlation by using the above summary results of 10 paired markets during the two subperiods. This is the study's fourth contribution to the literature because this simplified process makes us easily investigate the behavior of correlation, return spillover, and volatility spillover for five markets during two subperiods. Finally, regarding each pair of assets for two subperiods, the optimal asset allocation is investigated by examining the risk diversification of a portfolio with minimum risk whereas the optimal hedge strategy is also explored by examining the hedged cost and risk reduction of a hedged portfolio with minimum risk. This is the study's fifth contribution to the literature because most of the past literature related to spillover issues didn't discuss the issues of optimal asset allocation and the optimal hedge strategy. Hence, to mid the gap in the literature, this study utilizes both the network graphs and the results of a diagonal bivariate BEKK-GARCH model with two time-dummy variables to explore between five markets how the COVID-19 pandemic affects the correlation, return spillover, and volatility spillover. The five markets include the stock, oil, gold, currency, and cryptocurrency markets. In addition, the issues on the optimal asset allocation and the optimal hedge strategy are also explored in these five markets.

## Methodology

This study explores the variations of financial features related to a pair of market data during turbulent times such as the crisis of the COVID-19 pandemic. The financial features related to a pair of the market include correlation, return, and volatility spillovers. The paired markets contain the stock-oil, stock-gold, stock-currency, stock-cryptocurrency, oil-gold, oil-currency, oil-cryptocurrency, gold-currency, gold-cryptocurrency, and currency-cryptocurrency. Thus, this study utilizes a diagonal bivariate BEKK-GARCH model with the setting of two time-dummy variables to seize the financial features of interaction in the ten paired markets during the pre- and post-COVID-19 periods.

### A diagonal bivariate BEKK-GARCH-X model

The diagonal bivariate VAR(1)-BEKK-GARCH(1,1)-X model (hereafter, B-GARCH) is composed of the two-dimensional mean equation (**r**_**t**_) and two-dimensional variance-covariance equation (**H**_**t**_) with the normal distribution.^14^ The two-dimensional mean equation is expressed in the form of a bivariate vector autoregressive with lag one period [hereafter, VAR(1)], and is shown below.


(1) $$\begin{array}{l} {\text{r}}_{1,\text{t}}=\phi_{10}+\phi_{11}{\text{r}}_{1,\text{t}-1}+\phi_{12}{\text{r}}_{2,\text{t}-1}+\epsilon_{1,\text{t}} \end{array}$$

(2) $$\begin{array}{l} {\text{r}}_{2,\text{t}}=\phi_{20}+\phi_{21}{\text{r}}_{1,\text{t}-1}+\phi_{22}{\text{r}}_{2,\text{t}-1}+\epsilon_{2,\text{t}} \end{array}$$

where ${\text{r}}_{\text{t}}={\left(r_{1,t},r_{2,t}\right)}^{'}$ is a column vector of log returns and *r*_*i,t*_ = (ln *P*_*i,t*_ − *lnP*_*i,t*−1_) × 100 for *i* = 1, 2. *r*_*i,t*_ and *P*_*i,t*_ are the return and the close price of *i*^*th*^ asset of a pair of market data at time t, respectively. ϕ_10_, ϕ_11_, and ϕ_12_ are the parameters of the mean equation of the first asset whereas ϕ_20_, ϕ_21_ and ϕ_22_ are the parameters of the mean equation of the second asset. Parameters ϕ_12_ and ϕ_21_ are used to explore the return spillover between two markets. If parameter ϕ_12_ (respectively, ϕ_21_) is significant, then there exists a return spillover from the second (respectively, first) asset to the first (respectively, second) asset. $\varepsilon_{\text{t}}={\left(\varepsilon_{1,t},\varepsilon_{2,t}\right)}^{'}$ is a column vector of error terms, and its conditional distribution is assumed to follow the bivariate normal distribution with *E*_*t*−1_(**ε**_**t**_) = **0** and $E_{t-1}(\varepsilon_{\text{t}}\varepsilon_{\text{t}}^{'})={\text{H}}_{\text{t}};$ that is, **ε**_**t**_|**Ω**_**t−1**_ ~ *N*(**0**, **H**_**t**_). Subsequently, the two-dimensional variance-covariance equation, **H**_**t**_, is expressed as the form of the diagonal bivariate BEKK-GARCH(1,1)-X model and is expressed as follows:


(3) $$\begin{array}{l} {\text{h}}_{\text{t}}=\text{vech}\left({\text{H}}_{\text{t}}\right)={\left[{\text{h}}_{11,\text{t}},{\text{h}}_{12,\text{t}},{\text{h}}_{22,\text{t}}\right]}^{,} \end{array}$$

(4) $$\begin{array}{l} {\text{h}}_{11,\text{t}}=\omega_{1}+\alpha_{1}\varepsilon_{1,\text{t}-1}^{2}+\beta_{1}{\text{h}}_{11,\text{t}-1}+\nu_{12}{\text{h}}_{22,\text{t}-1} \end{array}$$

(5) $$\begin{array}{l} {\text{h}}_{12,\text{t}}=\omega_{12}+\alpha_{12}\varepsilon_{1,\text{t}-1}\varepsilon_{2,\text{t}-1}+\beta_{12}{\text{h}}_{12,\text{t}-1} \end{array}$$

(6) $$\begin{array}{l} {\text{h}}_{22,\text{t}}=\omega_{2}+\alpha_{2}\varepsilon_{2,\text{t}-1}^{2}+\beta_{2}{\text{h}}_{22,\text{t}-1}+\nu_{21}{\text{h}}_{11,\text{t}-1} \end{array}$$

where vech (**H**_**t**_) denotes the vech operator that stacks the “upper triangular” portion of a two-dimensional matrix **H**_**t**_ into a vector with a single column. *h*_11,*t*_ and *h*_22,*t*_ are the variances of the first and second assets of a pair of the market data at time t, respectively. ω_1_, α_1_, β_1_, and ν_12_ are the parameters of the variance equation for the first asset whereas ω_2_, α_2_, β_2_, and ν_21_ are the parameters of the variance equation for the second asset. *h*_12,*t*_ denotes the covariance between the returns of the two aforementioned assets at time t. ω_12_, α_12_, and β_12_ are the parameters of the covariance equation. Parameters ν_12_ and ν_21_ are used to explore the volatility spillover between two markets. If parameter ν_12_ (respectively, ν_21_) is significant, then there exists a volatility spillover from the second (respectively, first) asset to the first (respectively, second) asset. Notably, to seize the financial features of interaction on the six paired of markets during the pre- and post-COVID-19 periods, some parameters related to the correlation, return, and volatility spillovers must include two time-dummy variables, and then they are shown below.


$$\begin{array}{l} \phi_{12}=\phi_{12}^{\text{B}}{\cdot \text{d}}_{\text{t}}^{\text{B}}+\phi_{12}^{\text{A}}{\cdot \text{d}}_{\text{t}}^{\text{A}},\phi_{21}=\phi_{21}^{\text{B}}{\cdot \text{d}}_{\text{t}}^{\text{B}}+\phi_{21}^{\text{A}}{\cdot \text{ d}}_{\text{t}}^{\text{A}},\\ \nu_{12}=\nu_{12}^{\text{B}}{\cdot \text{d}}_{\text{t}}^{\text{B}}+\nu_{12}^{\text{A}}{\cdot \text{d}}_{\text{t}}^{\text{A}},\nu_{21}=\nu_{21}^{\text{B}}{\cdot \text{d}}_{\text{t}}^{\text{B}}+\nu_{21}^{\text{A}}{\cdot \text{ d}}_{\text{t}}^{\text{A}}, \end{array}$$

(7) $$\begin{array}{l} \omega_{12}=\omega_{12}^{\text{B}}{\cdot \text{d}}_{\text{t}}^{\text{B}}+\omega_{12}^{\text{A}}{\cdot \text{d}}_{\text{t}}^{\text{A}} \end{array}$$

where $d_{t}^{B}$ and $d_{t}^{A}$ are two time-dummy variables and can divide the study period into the subperiods Before and After the onset date of the COVID-19 pandemic (or pre- and post-COVID-19 subperiods). $d_{t}^{B}=1$ if *date*_*start*_ ≤ *t* < *date*_*covid*19_, and $d_{t}^{B}=$0 otherwise; $d_{t}^{A}=1$ if *date*_*covid*19_ ≤ *t* ≤ *date*_*end*_, and $d_{t}^{A}=$0 otherwise. *date*_*start*_ and *date*_*end*_ denote the start and end dates of the study sample, respectively. *date*_*covid*19_ represents the onset date of the COVID-19 pandemic. In addition, the parameters of this bivariate GARCH model are estimated by maximum likelihood (ML) optimizing numerically the bivariate Gaussian log-likelihood function. Hence, the log-likelihood function of the B-GARCH model can be written as follows:


$$\begin{array}{l} \text{L}\left(\Psi \right)=\overset{\text{m}}{\underset{\text{t}=1}{\sum }}\ln\left\{\text{f}\left({\text{r}}_{\text{t}}|\Omega_{\text{t}-1};\psi \right)\right\}=-\frac{\text{m}}{2}\text{ln}2\pi \end{array}$$

(8) $$\;-\frac{1}{2}\sum_{t=1}^{m}\left(\ln|H_{t}|+\varepsilon_{t}^{'}H_{t}^{-1}\varepsilon_{t}\right)$$

where $\Psi =[\phi_{10},\phi_{11},\;\phi_{12}^{B},\;\phi_{12}^{A},\;\phi_{20},\phi_{21}^{B},\;\phi_{21}^{A},\phi_{22},\omega_{1},\alpha_{1},\beta_{1},$ $\nu_{12}^{B},\nu_{12}^{A},\omega_{12}^{B},\;\omega_{12}^{A},\alpha_{12},\beta_{12},\omega_{2},\alpha_{2},\beta_{2},\;\nu_{21}^{B},\nu_{21}^{A}]$ is the vector of parameters to be estimated, m denotes the sample size of an estimate period; *f*(·) denotes the bivariate normal density, and Ω_*t*−1_ denotes the information set of all the observed returns up to time t-1. **r**_**t**_, **H**_**t**_ and **ε**_**t**_ are defined in Equations (1–6). Notably, the parameters with the superscript “B” (respectively, “A”) can seize the financial feature related to that parameter during the pre-COVID-19 (respectively, post-COVID-19) period. For instance, parameters $\phi_{12}^{B}$ and $\phi_{12}^{A}$ are utilized to explore whether there exists a return spillover from the second asset to the first asset during the pre- and post-COVID-19 periods, respectively.

### The optimal asset allocation and the optimal hedged strategy

This study used the Mean Dynamic Weight for the In-sample period (MDWI) approach of Su (41) or Kroner and Ng (42) to find the weight forecasts of the minimum variance portfolio (MVP) and then allocated the capital to the component assets to construct an optimal portfolio based on the minimum risk.^15^ Thus, the following technique of mathematical programming is used to determine the weights of the bivariate MVP.


$$\begin{array}{l} {\text{Minimize h}}_{\text{P},\text{t}}={\text{w}}_{1,\text{t}}^{2}{\cdot \text{h}}_{11,\text{t}}+{\text{w}}_{2,\text{t}}^{2}{\cdot \text{h}}_{22,\text{t}}+2{\text{w}}_{1,\text{t}}{\cdot \text{w}}_{2,\text{t}}{\cdot \text{ h}}_{12,\text{t}} \end{array}$$

(9) $$\begin{array}{l} \text{Subject to }\overset{\text{n}=2}{\underset{\text{i}=1}{\sum }}{\text{w}}_{\text{i},\text{t}}=1\text{and}-0.4\leq {\text{w}}_{\text{i},\text{t}}\leq 1.4\;for\text{ i}=1,2 \end{array}$$

Then, the in-sample weight forecast series of the two-component assets of the MVP are expressed as follows:


$$\begin{array}{l} {\text{w}}_{1,\text{t}}^{\text{MVP}}={\text{w}}_{1,\text{t}}=\frac{{\text{h}}_{22,\text{t}}-{\text{h}}_{12,\text{t}}}{{\text{h}}_{11,\text{t}}+{\text{h}}_{22,\text{t}}-2{\text{h}}_{12,\text{t}}},\text{ and }{\text{w}}_{2,\text{t}}^{\text{MVP}}={\text{w}}_{2,\text{t}} \end{array}$$

(10) $$\begin{array}{l} \;=1-{\text{w}}_{1,\text{t}}^{\text{MVP}} \end{array}$$

where $w_{1,t}^{MVP}$ and $w_{2,t}^{MVP}$ are the weights forecasts of the first and second component assets of the MVP at time t, respectively. Thus, for the MDWI approach, the weight forecasts of the two-component assets of the MVP are the mean values of the aforementioned in-sample weight forecast series of the two-component assets of the MVP, and they are expressed as follows:


$$\begin{array}{l} {\text{w}}_{1}^{\text{MVP}}\;=\frac{1}{\text{m}}\overset{\text{m}}{\underset{\text{t}=1}{\sum }}\frac{{\text{h}}_{22,\text{t}}-{\text{h}}_{12,\text{t}}}{{\text{h}}_{11,\text{t}}+{\text{h}}_{22,\text{t}}-2{\text{h}}_{12,\text{t}}},\text{ and }{\text{w}}_{2}^{\text{MVP}} \end{array}$$

(11) $$\begin{array}{ll} \;= & 1-{\text{w}}_{1}^{\text{MVP}} \end{array}$$

where $w_{1}^{MVP}$ and $w_{2}^{MVP}$ are the weights forecasts of the first and second component assets of the MVP, respectively. m is the sample size of the in-sample period or the estimate period, and it is set as 1,435 in this study. *h*_11,*t*_, *h*_22,*t*_, and *h*_12,*t*_ are defined in Equations (4–6). The return and variance of the MVP are expressed as follows:


(12) $$\begin{array}{l} {\text{R}}_{\text{t}}^{\text{MVP}}={\text{w}}_{1}^{\text{MVP}}.\;{\text{r}}_{1,\text{t}}+\left(1-{\text{w}}_{1}^{\text{MVP}}\right).\;{\text{r}}_{2,\text{t}} \end{array}$$

$$\begin{array}{l} {\text{h}}_{\text{t}}^{\text{MVP}}={\left({\text{w}}_{1}^{\text{MVP}}\right)}^{2}.\;{\text{h}}_{11,\text{t}}+{\left(1-{\text{w}}_{1}^{\text{MVP}}\right)}^{2}.\;{\text{h}}_{22,\text{t}} \end{array}$$

(13) $$\begin{array}{ll} \;+ & 2.{\text{w}}_{1}^{\text{MVP}}.\left(1-{\text{w}}_{1}^{\text{MVP}}\right).{\text{h}}_{12,\text{t}} \end{array}$$

Thus, the realized risk-adjusted returns of the MVP, $R_{t}^{a}$, are shown below.


(14) $$\begin{array}{l} {\text{R}}_{\text{t}}^{\text{a}}={\text{R}}_{\text{t}}^{\text{MVP}}/\sqrt{{\text{h}}_{\text{t}}^{\text{MVP}}} \end{array}$$

As to the optimal hedged strategy, two hedged portfolios, which are composed of two assets for a pair of market data, are constructed to explore the hedged issue and its risk reduction during the pre- and post-COVID-19 periods. For a pair of market data, the first hedged portfolio is constructed by a long position of one dollar on the first asset and a short position of β_1,*t*_ dollars on the second asset. Then, *R*_*p*1,*t*_ = *r*_1,*t*_ − β_1,*t*_*r*_2,*t*_ is the return of the first hedged portfolio, and $h_{p1,t}=\;h_{11,t}+\beta_{1,t}^{2}h_{22,t}-2\beta_{1,t}h_{12,t}$ is the variance of the first hedged portfolio. The second hedged portfolio is constructed by a long position of one dollar on the second asset and a short position of β_2,*t*_ dollars on the first asset. Then, *R*_*p*2,*t*_ = *r*_2,*t*_ − β_2,*t*_*r*_1,*t*_ is the return of the second hedged portfolio, and $h_{p2,t}=\;h_{22,t}+\beta_{2,t}^{2}h_{11,t}-2\beta_{2,t}h_{12,t}$ is the variance of the second hedged portfolio. Based on minimizing the risk of this hedged portfolio, the optimal hedge ratios β_1,*t*_ and β_2,*t*_ proposed by Kroner and Sultan (44) are expressed as follows:


(15) $$\begin{array}{l} \beta_{1,\text{t}}=\frac{{\text{h}}_{12,\text{t}}}{{\text{h}}_{22,\text{t}}},\beta_{2,\text{t}}=\frac{{\text{h}}_{12,\text{t}}}{{\text{h}}_{11,\text{t}}} \end{array}$$

where *h*_11,*t*_, *h*_22,*t*_, and *h*_12,*t*_ are defined in Equations (4–6). A high hedge ratio represents a high hedging cost. The hedging effectiveness (HE) or risk reduction effectiveness of the first and second hedged portfolios can be evaluated by examining the realized hedging errors, which are determined as follows (45):


(16) $$\begin{array}{l} {\text{HE}}_{1,\text{t}}^{\text{h}}=1-\frac{{\text{h}}_{\text{p}1,\text{t}}}{{\text{h}}_{11,\text{t}}},{\text{HE}}_{2,\text{t}}^{\text{h}}=1-\frac{{\text{h}}_{\text{p}2,\text{t}}}{{\text{h}}_{22,\text{t}}} \end{array}$$

where *h*_*p*1,*t*_ is the variance of the first hedged portfolio, and *h*_11,*t*_ is the variance of the unhedged portfolio in the case of the first hedged portfolio. *h*_*p*2,*t*_ is the variance of the second hedged portfolio, and *h*_22,*t*_ denotes the variance of the unhedged portfolio in the case of the first hedged portfolio. A higher HE ratio indicates greater hedging effectiveness in terms of the variance reduction of the portfolio, which thus implies that the associated investment method can be deemed as a better hedging strategy.

## Data and descriptive statistics

This study mainly explores how the crisis of the COVID-19 pandemic affects the interactions between the stock, oil, gold, currency, and cryptocurrency markets. Thus, the study data include the Dow Jones index, WTI crude oil, gold, Chinese yuan (CNY), Euro, Bitcoin, Ethereum, and Litecoin. The Dow Jones index is used to represent the stock market since it is one of the oldest and most commonly followed equity indices. Moreover, the CNY and Euro are used to represent the currency market because they are the top 10 most traded currencies in the world.^16^ Furthermore, Bitcoin, Ethereum, and Litecoin are used to represent the cryptocurrency market since they represent 76% of the cryptocurrency market capitalization (21). Notably, Bitcoin is the first blockchain-based cryptocurrency, and Litecoin and Ethereum are the two most well-known altcoins.^17^ In addition, the WTI crude oil is the main energy commodity whereas gold is a good anti-inflation asset. All the data have been downloaded from the Yahoo finance website, and they cover the period from August 20, 2015, to July 30, 2021. The study period is divided into the pre- and post-COVID-19 periods according to the onset date of the COVID-19 pandemic, which was March 11, 2020.^18^

Tables 2A–C list the basic descriptive statistics of the daily return of the study data during the overall period and the pre- and post-COVID-19 subperiods, respectively. From the data listed in the columns “Mean,” “SD,” or “*R*^*a*^,” the following values of the numbers are compared. Some interesting phenomena show up when the values of the mean return, standard deviation, or risk-adjusted return for the overall, and pre- and post-COVID-19 periods are compared at the same asset. First of all, regarding each asset, the greatest and smallest values of the mean return, standard deviation, or risk-adjusted return are all dispersed in the pre- or post-COVID-19 period. This result implies that the values of the mean return, standard deviation, or risk-adjusted return for the overall period are nearly the average values of the mean return, standard deviation, or risk-adjusted return for the pre- and post-COVID-19 periods. Secondly, except for the gold and CNY, the greatest values of the mean return or risk-adjusted return are all dispersed in the post-COVID-19 period. This result indicates that the QE implemented after the COVID-19 pandemic crisis increases the return and risk-adjusted return. Other interesting phenomena also show up when the values of the mean return, standard deviation, or risk-adjusted return for the eight assets are compared in the same period. First of all, regarding each period, the greatest values of the mean return, standard deviation, or risk-adjusted return are almost distributed at Ethereum, Bitcoin, and WTI, especially for Ethereum. Secondly, the smallest values of the mean return, standard deviation, or risk-adjusted return are almost distributed at the Euro, WTI, and CNY, especially for the Euro. The aforementioned results imply that Ethereum in the cryptocurrency market has the greatest return and the highest risk whereas the Euro in the currency market has the lowest return and the smallest risk. As the other descriptive statistics, they have almost the same features as those for most of the financial return series. For example, as shown by the coefficient of skewness and excess kurtosis, the distribution of returns is left-skewed or right-skewed and has a larger and thicker tail than that of the normal distribution. This result indicates that the return series isn't normally distributed, which is also confirmed by the J-B normality test statistics (36). Additionally, the return series exhibits linear dependence and the strong ARCH effect as shown by the Ljung-Box *Q*^2^(24) statistics for the squared returns. From the aforementioned findings, a GARCH family model is very suitable to seize the fat tails and time-varying volatility found in these asset return series.

**Table 2:** Descriptive statistics of the daily return for the overall, pre- and post-COVID-19 periods.

	**Mean**	**SD**	**R^a^**	**Max**.	**Min**.	**SK**	**KUR**	**J-B**	**Q^2^ (24)**
**A. The overall period**									
DowJones	0.0487	1.2441	0.0392	10.764	−13.84	−1.19^c^	24.530^c^	36,320^c^	2,150.8^c^
WTI	0.0415	4.5719	0.0090	42.583	−104.3	−7.55^c^	202.67^c^	2,469,737^c^	144.42^c^
Gold	0.0336	0.9289	0.0362	5.133	−5.26	−0.17^c^	4.38^c^	1,159.15^c^	285.46^c^
CNY	0.1893	5.7311	0.0330	51.141	−39.50	1.236^c^	11.63^c^	8,456.64^c^	160.76^c^
Euro	0.0048	0.4730	0.0103	3.064	−2.67	0.037	3.122	583.37^c^	175.55^c^
Bitcoin	0.3642	4.7657	0.0764	22.511	−46.47	−0.71^c^	9.52^c^	5,542.87^c^	48.54^c^
Ethereum	0.5282	7.4140	0.0712	50.968	−55.07	0.289^c^	6.70^c^	2,706.68^c^	91.21^c^
Litecoin	0.2603	6.7676	0.0384	53.984	−44.90	0.605^c^	11.03^c^	7,365.47^c^	115.29^c^
**B. The pre-COVID 19 period**									
DowJones	*0.0333*	*0.9605*	*0.0346*	4.967	−8.105	−1.01^c^	8.7087^c^	3,662^c^	1,187.9^c^
WTI	*−0.015*	*2.5431*	*−0.0059*	14.176	−28.13	−0.96^c^	15.715^c^	11,478.5^c^	102.92^c^
Gold	**0.0350**	*0.8228*	**0.0426**	4.196	−4.345	0.256^c^	2.853^c^	385.07^c^	130.43^c^
CNY	**0.2166**	**5.8695**	**0.0369**	51.141	−39.50	1.376^c^	13.063^c^	8,161.29^c^	141.52^c^
Euro	*0.0022*	**0.4841**	*0.0047*	3.064	−2.672	0.164^b^	3.269^c^	494.35^c^	122.74^c^
Bitcoin	*0.3232*	*4.5602*	*0.0708*	22.511	−23.87	−0.068	4.4481	906.89^c^	116.62^c^
Ethereum	*0.4615*	**7.4606**	*0.0618*	50.968	−29.18	0.758^c^	5.282^c^	1,383.4^c^	117.74^c^
Litecoin	*0.2430*	*6.6551*	*0.0365*	53.984	−39.50	1.438^c^	11.640^c^	6,583.6^c^	119.52^c^
**C. The post-COVID 19 period**									
DowJones	**0.0993**	**1.8969**	**0.0523**	10.764	−13.84	−1.07^c^	16.624^c^	3,934^c^	674.0^c^
WTI	**0.2270**	**8.2603**	**0.0274**	42.583	−104.3	−5.47^c^	80.676^c^	92,801.02^c^	29.31
Gold	*0.0290*	**1.2142**	*0.0239*	5.133	−5.26	−0.59^c^	3.852^c^	227.52^c^	99.92^c^
CNY	*0.1000*	*5.2604*	*0.0190*	27.747	−18.02	0.566^c^	3.716^c^	211.37^c^	15.37
Euro	0.0133	*0.4353*	**0.0307**	1.738	−1.77	−0.52^c^	2.243^c^	85.73^c^	93.57^c^
Bitcoin	**0.4985**	**5.3886**	**0.0925**	19.152	−46.47	−2.00^c^	17.736^c^	4,629.89^c^	18.03
Ethereum	**0.7466**	*7.2664*	**0.1027**	32.497	−55.07	−1.37^c^	12.323^c^	2,231.56^c^	32.23
Litecoin	**0.3169**	**7.1335**	**0.0444**	23.695	−44.90	−1.62^c^	9.592^c^	1,435.75^c^	33.99^a^

1. The superscripts a, b, and c denote significantly at the 10, 5, and 1% levels, respectively. 2. Mean denotes the mean return and SD represents the standard deviation of return. *R*^*a*^ denotes the realized risk-adjusted returns. 3. SK and KUR denote the skewness and excess kurtosis, respectively. 4. J-B statistics are based on Jarque and Bera (36) and are asymptotically chi-squared-distributed with 2 degrees of freedom. 5. *Q*^2^(24) statistics are asymptotically chi-squared-distributed with 24 degrees of freedom. 6. The bold and italic fonts in columns “Mean,” “SD,” or “*R*^*a*^” respectively denote the largest and smallest values of the mean, standard deviation, or the realized risk-adjusted returns when the values of mean, standard deviation, or the realized risk-adjusted returns for the overall, pre- and post-COVID-19 periods are compared each other at the same asset. 7. The shade and underline fonts in columns “Mean,” “SD” or “*R*^*a*^” respectively denote the largest and smallest values of the mean, standard deviation, or the realized risk-adjusted returns when the values of mean, standard deviation, or the realized risk-adjusted returns for all eight assets are compared each other in the same period. 8. The date of COVID-19 occurring is March 11, 2020. 9. The overall period is from August 20, 2015, to July 30, 2021.

**Figure 1:**
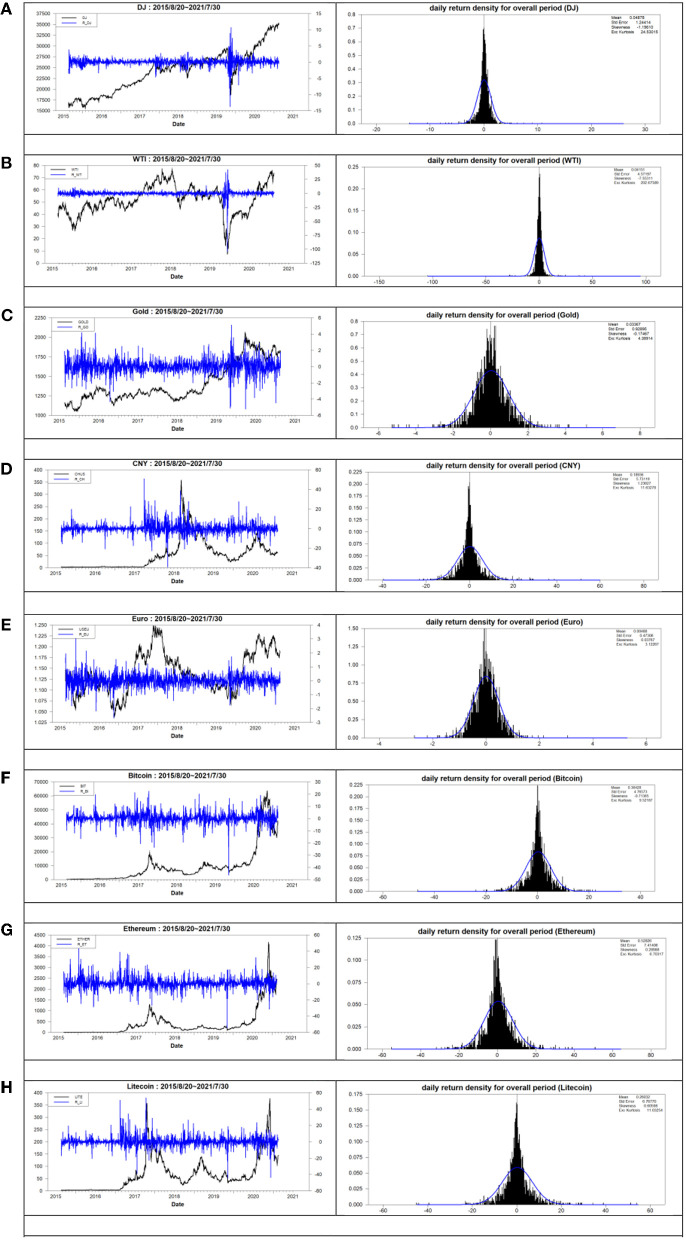
The price level, return, and daily return density for the overall period. **(A)** DowJones, **(B)** WTI, **(C)** Gold, **(D)** CNY, **(E)** Euro, **(F)** Bitcoin, **(G)** Ethereum, and **(H)** Litcoin.

The left panel of Figure 1 illustrates both the trends of price levels and returns for the eight assets during the overall period. The right panel of Figure 1 illustrates the daily return density for the eight assets during the overall period. From the left panel of Figure 1, the price of assets underwent a rapid rise after the crisis of the COVID-19 pandemic owing to the QE implemented after the aforementioned crisis. The volatility clustering occurs significantly during the overall period. From the right panel of Figure 1, the distribution of returns has a larger and thicker tail than that of the normal distribution. The aforementioned phenomenon is the same as that from the aforementioned analysis in Table 2.

## Empirical results

*Via* observing the variations of the financial features related to a pair of market data during the pre- and post-COVID-19 periods, this study explores how the crisis of the COVID-19 pandemic affects the interactions among the stock, oil, gold, currency, and cryptocurrency markets. Hence, the pairs of markets contain stock-oil (1), stock-gold (1), stock-currency (2), stock-cryptocurrency (3), oil-gold (1), oil-currency (2), oil-cryptocurrency (3), gold-currency (2), gold-cryptocurrency (2), and currency-cryptocurrency (6), totaling 23 pairs of assets.^19^ The financial features related to a pair of markets include correlation, return and volatility spillovers. Regarding the aforementioned ten paired markets, this study also explores the optimal asset allocation and optimal hedged strategy in the era of the COVID-19 pandemic.

### The results of the variations in the interactions of the ten paired markets in the era of the COVID-19 pandemic

In this subsection, we observe the significant situation of the parameters related to correlation, return, and volatility spillovers for the pre- and post-COVID-19 periods on the B-GARCH model to study the variations of financial features of interactions owing to the crisis of the COVID-19 pandemic. The parameters related to the correlation are $\omega_{12}^{B}$ and $\omega_{12}^{A}$; the parameters related to the return and volatility spillovers include “$\phi_{12}^{B},\;\phi_{12}^{A},\;\phi_{21}^{B},\;$and $\phi_{21}^{A}$” and “$\nu_{12}^{B},\;\nu_{12}^{A},\;\nu_{21}^{B},\;$and $\nu_{21}^{A}$,” respectively. Therefore, the empirical results of the parameters related to the interactions for the 23 pairs of assets or ten paired markets are listed in Table 3. The ten paired markets are the stock-oil, stock-gold, stock-currency, stock-cryptocurrency, oil-gold, oil-currency, oil-cryptocurrency, gold-currency, gold-cryptocurrency, and currency-cryptocurrency. However, it is very difficult to discuss the issues addressed in this study *via* the results listed in Table 3. Thus, regarding the pre- and post-COVID-19 periods, we summarize the results of three types of interactions for all the 10 paired markets in Table 3 into Table 4.

**Table 3:** The results of three types of interactions for all the 10 paired markets.

	**Stock-Oil**	**Stock-Gold**	**Stock-Currency**		**Stock-Cryptocurrency**			**Oil-Gold**
	**dj-wt**	**dj-go**	**dj-ch**	**dj-eu**	**dj-bi**	**dj-et**	**dj-li**	**wt-go**
$\phi_{12}^{B}$	−0.0067 (0.008)	−0.0365 (0.026)	0.0060 (0.002)^b^	0.0807 (0.036)^b^	0.0065 (0.003)^a^	0.0020 (0.002)	−0.0021 (0.002)	0.0278 (0.073)
$\phi_{21}^{B}$	0.1858 (0.072)^b^	−0.0279 (0.023)	0.1121 (0.142)	−0.0010 (0.013)	0.4624 (0.117)^c^	0.4998 (0.168)^c^	0.5831 (0.172)^c^	0.0081 (0.008)
$\phi_{12}^{A}$	0.0177 (0.012)	−0.0185 (0.026)	−0.0204 (0.010)^b^	−0.1593 (0.107)	0.0033 (0.010)	0.0070 (0.006)	0.0016 (0.006)	−0.1407 (0.117)
$\phi_{21}^{A}$	0.1845 (0.122)	−0.0046 (0.014)	0.0315 (0.166)	0.0883 (0.012)^c^	0.0011 (0.155)	0.0248 (0.186)	−0.0035 (0.176)	0.0053 (0.010)
$\nu_{12}^{B}$	−0.0000 (0.001)	−0.0068 (0.005)	0.0001 (0.000)	−0.0793 (0.037)^b^	−0.0007 (0.000)^c^	−0.0001 (0.000)^b^	−0.0003 (0.000)^c^	0.2720 (0.098)^c^
$\nu_{21}^{B}$	0.2287 (0.073)^c^	0.0024 (0.004)	−0.0114 (0.089)	0.0005 (0.000)	0.3676 (0.115)^c^	1.5460 (0.387)^c^	0.3039 (0.203)	−0.0000 (0.000)
$\nu_{12}^{A}$	0.0006 (0.000)	0.0003 (0.001)	0.0009 (0.000)^a^	0.1261 (0.135)	0.0001 (0.000)	0.0001 (0.000)	−0.0002 (0.000)	0.1408 (0.061)^b^
$\nu_{21}^{A}$	0.2365 (0.092)^b^	−0.0003 (0.000)	0.1988 (0.098)^b^	−0.0001 (0.000)	0.0581 (0.064)	−0.1074 (0.070)	−0.1464 (0.040)^c^	−0.0001 (0.000)^c^
$\omega_{12}^{B}$	0.0187 (0.007)^c^	0.0044 (0.010)	0.1160 (0.143)	−0.0163 (0.010)	−0.0042 (0.009)	0.0190 (0.015)	−0.0018 (0.016)	0.0380 (0.029)
$\omega_{12}^{A}$	0.0319 (0.013)^b^	−0.0587 (0.071)	0.2059 (0.400)	0.0789 (0.024)^c^	0.0427 (0.028)	0.0650 (0.037)^a^	0.0820 (0.053)	0.0529 (0.055)
	**Oil-Currency**		**Oil-Cryptocurrency**			**Gold-Currency**		
	**wt-ch**	**wt-eu**	**wt-bi**	**wt-et**	**wt-li**	**go-ch**	**go-eu**	**Sum (23)**
$\phi_{12}^{B}$	0.0038 (0.008)	−0.0611 (0.1198)	−0.0021 (0.012)	−0.0018 (0.009)	−0.0112 (0.009)	0.0033 (0.003)	0.1218 (0.045)^c^	√ (4+, 1–)
$\phi_{21}^{B}$	0.0312 (0.051)	4.8e−3 (5.0e-3)	−0.0603 (0.037)	0.0722 (0.089)	−0.0437 (0.051)	0.1355 (0.123)	−0.0195 (0.015)	√ (4+, 1–)
$\phi_{12}^{A}$	−0.0203 (0.026)	−0.3245 (0.2592)	0.0539 (0.020)^c^	0.0523 (0.014)^c^	0.0379 (0.013)^c^	0.0022 (0.010)	0.2007 (0.145)	√ (3+, 1–)
$\phi_{21}^{A}$	−0.0052 (0.035)	4.0e−3 (4.0e-3)	0.0464 (0.037)	0.0605 (0.046)	0.0278 (0.040)	0.0867 (0.214)	0.0453 (0.015)^c^	√ (2+, 3–)
$\nu_{12}^{B}$	−0.0022 (0.000)^c^	−0.3760 (0.2530)	−0.0028 (0.000)^c^	−0.0009 (0.000)	−0.0010 (0.000)^c^	−0.0003 (0.000)^c^	−0.0188 (0.026)	–(2+, 11–)
$\nu_{21}^{B}$	−0.0643 (0.011)^c^	−9.4e-6 (8.6e-5)	−0.0649 (0.006)^c^	0.2885 (0.082)^c^	−0.1007 (0.010)^c^	−2.1056 (0.549)^c^	0.0007 (0.001)	? (7+, 7–)
$\nu_{12}^{A}$	−0.0034 (0.001)^b^	−0.4156 (0.4453)	−0.0029 (0.001)^b^	−0.0015 (0.000)	−0.0015 (0.000)^a^	0.0009 (0.000)^c^	0.1213 (0.082)	√ (7+, 3–)
$\nu_{21}^{A}$	0.0017 (0.002)	−7.6e-6 (5.4e-6)	0.0010 (0.001)	0.0009 (0.002)	−0.0029 (0.000)^c^	−0.5010 (0.287)^a^	−0.0004 (0.000)	√ (2+, 8–)
$\omega_{12}^{B}$	−0.0107 (0.017)	0.0282 (0.0214)	0.0047 (0.006)	1.3236 (0.762)^a^	1.0298 (0.702)	−0.0051 (0.048)	0.0104 (0.002)^c^	√ (3+)
$\omega_{12}^{A}$	0.0667 (0.058)	0.0707 (0.0400)^a^	−0.0076 (0.016)	1.1784 (1.235)	2.0265 (1.522)	0.0602 (0.149)	0.0154 (0.003)^c^	√ (9+)
	**Gold-Cryptocurrency**		**Currency-Cryptocurrency**					
	**go-et**	**go-li**	**ch-bi**	**ch-et**	**ch-li**	**eu-bi**	**eu-et**	**eu-li**
$\phi_{12}^{B}$	0.0018 (0.003)	−0.0022 (0.003)	−0.0461 (0.027)^a^	0.0198 (0.012)	−0.0175 (0.015)	−0.0008 (0.002)	0.0019 (0.001)	0.0007 (0.001)
$\phi_{21}^{B}$	−0.1243 (0.235)	−0.1969 (0.138)	−0.0239 (0.022)	0.0173 (0.025)	−0.0005 (0.028)	−0.4442 (0.146)^c^	−0.4571 (0.365)	−0.4242 (0.298)
$\phi_{12}^{A}$	0.0056 (0.008)	0.0081 (0.009)	0.0572 (0.045)	0.0296 (0.036)	0.0415 (0.033)	0.0055 (0.004)	0.0048 (0.002)	0.0045 (0.003)
$\phi_{21}^{A}$	−0.0877 (0.298)	−0.1287 (0.305)	−0.0836 (0.045)^a^	−0.1409 (0.061)^b^	−0.1100 (0.060)^a^	−0.0822 (0.555)	−1.1027 (0.679)	0.2288 (0.843)
$\nu_{12}^{B}$	0.0001 (0.000)	−0.0000 (0.000)^b^	0.0189 (0.013)	−0.0117 (0.002)^c^	−0.0122 (0.001)^c^	−0.0000 (0.000)	0.0000 (0.000)^b^	0.0000 (0.000)
$\nu_{21}^{B}$	3.7669 (1.657)^b^	−3.6602 (0.070)^c^	0.0158 (0.010)	−0.0130 (0.014)	0.0182 (0.010)^a^	−4.0122 (0.052)^c^	20.3682 (7.573)^c^	−6.6027 (1.575)^c^
$\nu_{12}^{A}$	0.0007 (0.000)^b^	0.0002 (0.000)^b^	0.0266 (0.015)^a^	−0.0023 (0.004)	−0.0054 (0.005)	0.0000 (0.000)	0.0000 (0.000)^a^	0.0000 (0.000)
$\nu_{21}^{A}$	0.8376 (0.688)	−1.5903 (0.211)^c^	0.0196 (0.012)	−0.0538 (0.023)^b^	−0.0078 (0.016)	−4.1559 (0.329)^c^	13.4004 (8.642)	−9.7810 (2.100)^c^
$\omega_{12}^{B}$	0.0913 (0.082)	0.0127 (0.015)	0.4129 (0.560)	−1.1339 (1.963)	0.0546 (0.161)	0.0138 (0.014)	0.0768 (0.087)	−0.1177 (0.123)
$\omega_{12}^{A}$	0.2608 (0.295)	0.0843 (0.076)	1.0558 (1.051)	7.0116 (3.260)^b^	0.4365 (0.424)	0.1779 (0.026)^c^	0.5102 (0.288)^a^	1.1506 (0.254)^c^

1. “dj” denotes the Dow Jones index in the US stock market; “wt” and “go” represent the WTI crude oil and gold in the commodity market, respectively; “ch” and “eu” denote the Chinese yuan (CNY) and Euro in the currency market, respectively, and “bi,” “et,” and “li” denote the abbreviations of Bitcoin, Ethereum, and Litecoin in the cryptocurrency market, respectively. 2. The superscripts a, b, and c on a parameter estimate denote the parameter estimate is significant at the 10, 5, and 1% levels, respectively. 3. The superscript ‘B' on the parameters ‘$\phi_{12}^{B}$, $\phi_{21}^{B}$, $\nu_{12}^{B}$, $\nu_{21}^{B}$, and $\omega_{12}^{B}$' and the superscript ‘A' on the parameters ‘$\phi_{12}^{A}$, $\phi_{21}^{A}$, $\nu_{12}^{A}$, $\nu_{21}^{A}$, and $\omega_{12}^{A}$' denote that the parameters are corresponding to the pre-COVID-19 and post-COVID-19 periods, respectively. 4. The symbol “+” (respectively, “–”) in column “Sum” denotes a financial feature corresponding to a parameter that is positively (respectively, negatively) significant when taking all ten paired markets as a whole. That is, the total number of pairs of assets that have a significantly positive or negative parameter value is greater than half of the sample size, respectively. In addition, the numbers inside a bracket beside the aforementioned symbol “+” or “–” denote the total number of pairs of assets that have a significantly positive or negative parameter value, respectively. 5. The symbol “√” in column “Sum” denotes a financial feature corresponding to a parameter that is slightly significant when taking all ten paired markets as a whole. That is, the total number of pairs of assets that have a significantly positive or negative parameter value is less than half of the sample size. In addition, the numbers inside a bracket beside the aforementioned symbol “√” denote the total number of pairs of assets that have a significantly positive or negative parameter value, respectively. 6. The symbol “?” in column “Sum” denotes a financial feature corresponding to a parameter that is significant when taking all ten paired markets as a whole. However, whether this financial feature is significantly positive or negative cannot be concluded because the total number of pairs of assets that have a significantly positive and negative parameter value is equal, respectively.

**Table 4:** The summary results of three types of interactions for all the 10 paired markets.

	**Stock-Oil**	**Stock-Gold**	**Stock-Currency**			**Stock-Cryptocurrency**				**Oil-Gold**
	**dj-wt**	**dj-go**	**dj-ch**	**dj-eu**	**S**	**dj-bi**	**dj-et**	**dj-li**	**S**	**wt-go**
**A. The return spillover**										
Pre	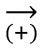	×	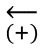	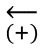	←	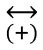	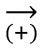	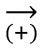	→	×
Post	×	×	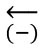	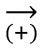	?	×	×	×	×	×
**B. The volatility spillover**										
Pre	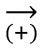	×	×	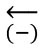	√(←)	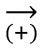	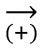	×	→	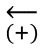
Post	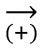	×	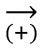	×	√(→)	×	×	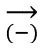	√(→)	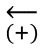
**C. The correlation**										
Pre	+	×	×	×	×	×	×	×	×	×
Post	+	×	×	+	√(+)	×	+	×	√(+)	×
**D. The type of interaction affected by the COVID-19 pandemic**										
Interaction	Return	×			×				Return	×
	**Oil-Currency**			**Oil-Cryptocurrency**				**Gold-Currency**		
	**wt-ch**	**wt-eu**	**S**	**wt-bi**	**wt-et**	**wt-li**	**S**	**go-ch**	**go-eu**	**S**
**A. The return spillover**										
Pre	×	×	×	×	×	×	×	×	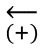	√(←)
Post	×	×	×	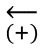	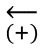	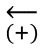	←	×	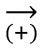	√(→)
**B. The volatility spillover**										
Pre	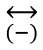	×	√(↔)	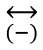	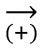	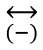	↔	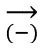	×	√(→)
Post	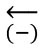	×	√(←)	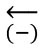	×	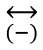	←	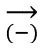	×	√(→)
**C. The correlation**										
Pre	×	×	×	×	+	×	√(+)	×	+	√(+)
Post	×	+	√(+)	×	×	×	×	×	+	√(+)
**D. The type of interaction affected by the COVID-19 pandemic**										
Interaction			×				Return			×
	**Gold-Cryptocurrency**			**Currency-Cryptocurrency**						
	**go-et**	**go-li**	**S**	**ch-bi**	**ch-et**	**ch-li**	**eu-bi**	**eu-et**	**eu-li**	**S**
**A. The return spillover**										
Pre	×	×	×	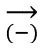	×	×	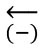	×	×	√(?)
Post	×	×	×	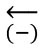	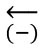	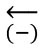	×	×	×	√(→)
**B. The volatility spillover**										
Pre	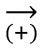	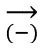	→	×	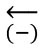	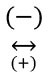	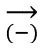	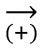	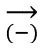	→
Post	×	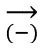	√(→)	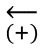	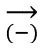	×	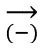	×	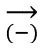	→
**C. The correlation**										
Pre	×	×	×	×	×	×	×	×	×	×
Post	×	×	×	×	+	×	+	+	+	+
**D. The type of interaction affected by the COVID-19 pandemic**										
Interaction			×							Correlation

1. “dj” denotes the DowJones index in the US stock market; “wt” and “go” represent the WTI crude oil and gold in the commodity market, respectively; “ch” and “eu” denote the Chinese yuan (CNY) and Euro in the currency market, respectively, and “bi,” “et,” and “li” denote the abbreviations of Bitcoin, Ethereum, and Litecoin in the cryptocurrency market, respectively. 2. The symbol “ × ” represents that the interaction (correlation and return and volatility spillovers) of a pair of assets does not exist. The symbol “+” in C denotes that the correlation is positive significantly for a pair of assets. For example, the value of parameter $\omega_{12}^{B}$ or $\omega_{12}^{A}$ in Table 3 is significantly positive. 3. The symbol “ → ” in A,B denotes that the spillover from the first asset to the second asset significantly exists for a pair of assets if the value of parameter “$\phi_{21}^{B}$ or $\phi_{21}^{A}$” for the return spillover in Table 3 is significant, so is the case for parameter “$\nu_{21}^{B}$ or $\nu_{21}^{A}$” for the volatility spillover in Table 3. 4. The symbol “←” in A, B denotes that the spillover from the second asset to the first asset significantly exists for a pair of assets if the value of parameter “$\phi_{12}^{B}$ or $\phi_{12}^{A}$” for the return spillover in Table 3 is significant, so is the case for parameter “$\nu_{12}^{B}$ or $\nu_{12}^{A}$” for the volatility spillover in Table 3. 5. The symbol “+” (respectively, “–”) inside the bracket underneath the symbol “ → ” or “←” in panels A-B denotes that the spillover is significantly positive (respectively, negative). 6. The symbol “ × ” in column “S” underneath a paired market data denotes a specific financial feature corresponding to a parameter that does not exist for that paired market data. That is, the total number of pairs of assets that have a significantly positive or negative parameter value is zero, respectively. 7. The symbol “**→**” (or, “**←**”) in column “S” underneath a paired market data denotes a spillover effect significantly exist on that paired market data. That is, the total number of pairs of assets that have a significant spillover effect is greater than half of the sample size, respectively. 8. The symbol “√” in column “S” underneath a paired market data denotes a financial feature corresponding to a parameter that is slightly significant on that paired market data. That is, the total number of pairs of assets that have a significantly positive and negative parameter value is less than half of the sample size. In addition, the symbols inside a bracket beside the aforementioned symbol “√” record the most state of significant financial features for that paired market data. 9. The symbol “?” in column “S” underneath a paired market data denotes a financial feature corresponding to a parameter that is significant on that paired market data. However, whether this financial feature such as a spillover “**→**” or spillover “**←**” cannot be concluded because the total number of pairs of assets that have a significant spillover “**→**” and spillover “**←**” are equal, respectively.

Subsequently, we take an example of the dj-bi pair of data in Table 3 to illustrate the above summary process. Parameters $\phi_{12}^{B}$ (0.0065) and $\phi_{21}^{B}$ (0.4624) are significantly positive. This result indicates that there exists a bidirectional and positive return spillover between the Dow Jones and bitcoin during the pre-COVID-19 period. We record this result as the symbol “$\overset{↔}{\left(+\right)}$” in the row “pre” and the column “dj-bi” underneath “stock-cryptocurrency” in the Table 4A. Moreover, parameters $\phi_{12}^{A}$ (0.0033) and $\phi_{21}^{A}$ (0.0011) are not significant. This result indicates that no return spillover exists in the dj-bi pair of assets during the post-COVID-19 period. We record this result as the symbol “**×**”in the row “post” and the column “dj-bi” underneath “stock-cryptocurrency” in Table 4A. Furthermore, parameters $\nu_{12}^{B}$ (−0.0007) and $\nu_{21}^{B}$ (0.3676) are significantly negative and positive, respectively. This result indicates that there exists a positive volatility spillover from Dow Jones to bitcoin owing to the value “0.3676” of parameter “$\nu_{21}^{B}$” being positive. Conversely, there also exists a negative volatility spillover from bitcoin to Dow Jones owing to the value “−0.0007” of parameter “$\nu_{12}^{B}$” being negative. However, the value “−0.0007” nearly approaches zero, then we neglect it. Thus, we record these results as the symbol “$\vec{\left(+\right)}$”in the row “pre” and the column “dj-bi” underneath “stock-cryptocurrency” in the Table 4B. At the same inference process, we complete the summary process for the remaining pairs of assets.

Finally, for some paired markets, which include more than one pair of data, a rule is made to determine whether a financial feature of the interactions is significant, slightly significant, or insignificant. The above-paired markets include the stock-currency, stock-cryptocurrency, oil-currency, oil-cryptocurrency, gold-currency, gold-cryptocurrency, and currency-cryptocurrency. First of all, regarding the stock-cryptocurrency paired market, the symbol “$\overset{\to }{\left(+\right)}$” appears in the row “pre” and the columns “dj-bi” and “dj-et” in Table 4B. This result indicates that the total number of pairs of assets that have a significant volatility spillover equals 2, which is greater than half of the sample size (2/3). We record this result as the symbol “**→**” in the row “pre” and the column “S” underneath “stock-cryptocurrency” in the Table 4B. This result signifies that there significantly exists a volatility spillover from the stock market to the cryptocurrency market during the pre-COVID-19 period. Secondly, the symbol “$\overset{\to }{\left(-\right)}$” only appears in the row “post” and the column “dj-li” in Table 4B. This result indicates that the total number of pairs of assets that have a significant volatility spillover equals 1, which is less than half of the sample size (1/3). We record this result as the symbol “√(→)” in the row “post” and the column “S” underneath “stock-cryptocurrency” in Table 4B. This result represents that a volatility spillover from the stock market to the cryptocurrency market is slightly significant during the post-COVID-19 period. Thirdly, the symbol “**×**” appears in the row “pre” and all the columns “dj-bi,” “dj-et,” and “dj-li” in Table 4C. We record this result as the symbol “**×**” in the row “pre” and the column “S” underneath “stock-cryptocurrency” in Table 4C. This result indicates that no significant correlation exists between the stock market and cryptocurrency market during the pre-COVID-19 period. Fourthly, regarding the stock-currency paired market, the symbols “$\overset{\leftarrow }{\left(-\right)}$” and “$\vec{\left(+\right)}$” appear in the row “post” and respectively the columns “dj-ch” and “dj-eu” in Table 4A. This result indicates that the total number of pairs of assets that have a significant return spillover equals 2, which is greater than half of the sample size (2/2). However, the total number of pairs of assets that have a significant return spillover “**→**” is equal to the total number of pairs of assets that have a significant return spillover “**←**”. Then, we can't conclude the direction of this return spillover is “**→**” or “**←**”. Then, the symbol “?” is recorded in the row “post” and the column “S” underneath “stock-currency” in Table 4A. This result indicates that there exists a return spillover between the stock market and the currency market but we don't know whether this spillover is from the stock market to the currency market or from the currency market to the stock market. At the same inference process, we complete the summary results for the remaining paired markets such as the oil-currency, oil-cryptocurrency, gold-currency, gold-cryptocurrency, and currency-cryptocurrency paired stock markets.

**Figure 2:**
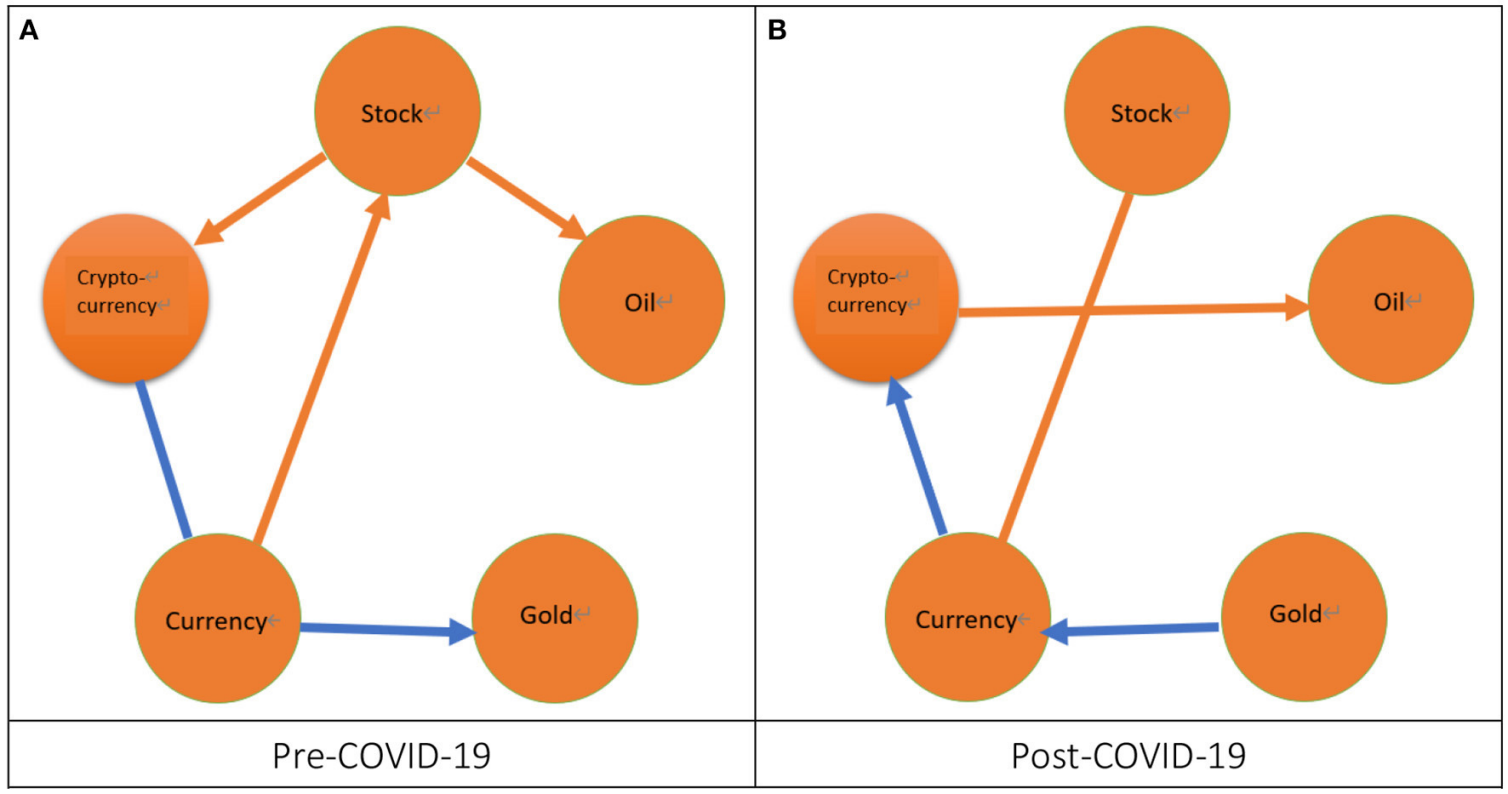
The network of return spillover. 1. The orange arrow from a specific node to another node denotes a significant return spillover from a market represented by this specific node to another market represented by another node. 2. The blue arrow from a specific node to another node represents a slightly significant return spillover from a market represented by this specific node to another market represented by another node. 3. The orange line connected by two nodes denotes a significant return spillover between two markets represented by these two nodes but the direction of this spillover is uncertain. 4. The blue line connected by two nodes denotes a slightly significant return spillover between two markets represented by these two nodes but the direction of this spillover is uncertain. **(A)** Pre-COVID-19 and **(B)** Post-COVID-19.

**Figure 3:**
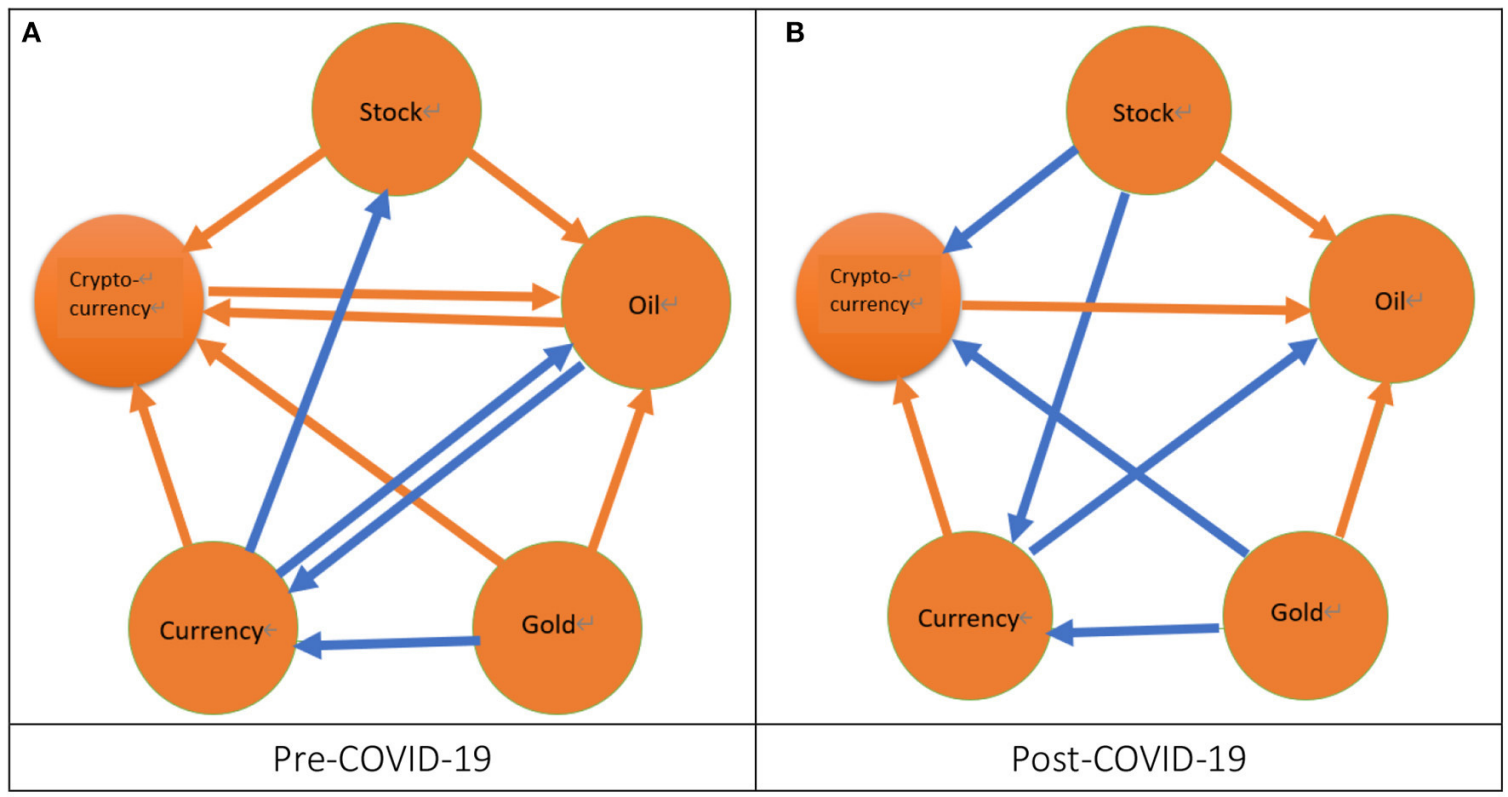
The network of volatility spillover. 1. The orange arrow from a specific node to another node denotes a significant volatility spillover from a market represented by this specific node to another market represented by another node. 2. The blue arrow from a specific node to another node represents a slightly significant volatility spillover from a market represented by this specific node to another market represented by another node. **(A)** Pre-COVID-19 and **(B)** Post-COVID-19.

**Figure 4:**
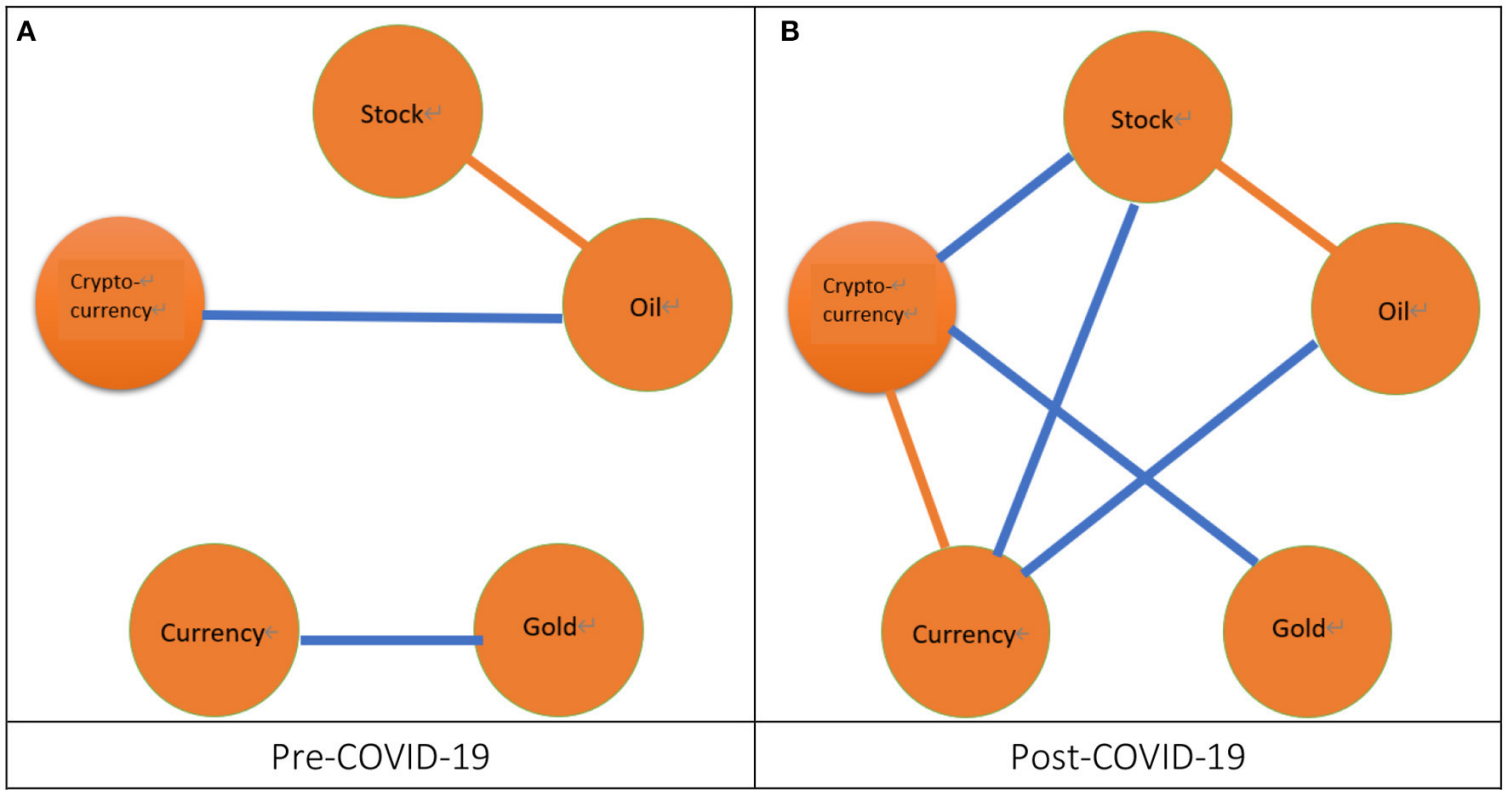
The network of correlation. 1. The orange line connected by two nodes denotes a significant correlation between two markets represented by these two nodes. 2. The blue line connected by two nodes denotes a slightly significant correlation between two markets represented by these two nodes. **(A)** Pre-COVID-19 and **(B)** Post-COVID-19.

However, it is still difficult to discuss the issues addressed in this study *via* the results listed in Table 4. Thus, we follow Umar et al. (32) to construct the network graphs of return spillover, volatility spillover, and correlation by using the summary results of 10 paired markets listed in column “S” in Table 4.^20^ We will take an example of Figure 2A to illustrate how to get a network graph of return spillover during the pre-COVID-19 period by using the summary results in the row “pre” and the column “S” underneath each paired market in Table 4A. Subsequently, we compare the significant results of this study with those of literature, which are listed in the column “Findings” in Table 1. Figures 2–**4** depicts the network graphs of return spillover, volatility spillover, and correlation for the ten paired markets, respectively. First of all, from Figure 2A, we can observe that during the pre-COVID-19 period there are three cases of significant return spillovers. One return spillover is from the currency market to the stock market, which is consistent with Su (14), and Sui and Sun (15), but is different from Su (19), Yang and Doong (12), Kumar (13), and Erdogan et al. (16).^21^ The other two cases of return spillover are from the stock market to both the oil and cryptocurrency markets, which is consistent with the pre-SB period in Su (19) and the FTSE-bitcoin pair of indices in Uzonwanne (18) but is different from Yousaf and Hassan (9).^22^ The above results in Figure 2A are summarized from the results in the row “pre” and the columns “dj-wt” underneath “stock-oil” and “S” underneath “stock-currency” and “stock-cryptocurrency” in Table 4A. In the same inference process, two slightly significant return spillovers in the row “pre” and the columns “S” underneath “gold-currency” and “currency-cryptocurrency” in Table 4A are marked in Figure 2A. One case of return spillover from the currency market to the gold market is slightly significant. Conversely, another case of return spillover between the currency market and the cryptocurrency market is also slightly significant. However, we don't know whether this spillover is from the currency market to the cryptocurrency market or from the cryptocurrency market to the currency market. From Figure 2B, we find that there are two cases of significant return spillovers during the post-COVID-19 period. One case of return spillover is from the cryptocurrency market to the oil market, which is consistent with Yousaf et al. (22).^23^ Another case of return spillover exists between the stock market and the currency market but the direction of this spillover is uncertain. This result is similar to Su (19), which exists a return spillover from the stock market to the currency market. In addition, two cases of return spillovers are slightly significant. One spillover is from the gold market to the currency market and another spillover is from the currency market to the cryptocurrency market. Subsequently, we compare the results of return spillover for the pre- and post-COVID-19 periods. We find that, after the COVID-19 pandemic, the significant cases decrease because the significant cases of spillover for the pre-COVID-19 period are three but two for the post-COVID-19 period. Moreover, after the COVID-19 pandemic, two cases of spillover from the stock market to both the oil and cryptocurrency markets completely disappeared but one spillover case from the cryptocurrency market to the oil market appears.

Secondly, from Figure 3, we can observe that irrespectively of the pre-COVID-19 period or the post-COVID-19 period, the volatility spillover within five markets is closely related as compared with the phenomena found from the return spillover. From Figure 3A, we find that during the pre-COVID-19 period there exist seven cases of significant volatility spillovers. That is, two cases of spillover are from the stock market to both the oil and cryptocurrency markets, which is consistent with Gomez-Gonzalez et al. (46), Jebabli et al. (47), the pre-SB period in Su (19), and Uzonwanne (18).^24^ Another two cases of spillover are from the gold market to both the oil and cryptocurrency markets, which is consistent with Yousaf et al. (22).^25^ Moreover, one case of spillover is from the currency market to the cryptocurrency market and another case of bidirectional spillover exists between the oil market and the cryptocurrency market, which is similar to Yousaf et al. (22).^26^ We also find that there exist four cases of significant volatility spillovers during the post-COVID-19 period as shown in Figure 3B. That is, three cases of spillover are from the stock, gold, and cryptocurrency markets to the oil market, which is consistent with Gomez-Gonzalez et al. (46), Jebabli et al. (47), the pre-SB period in Su (19), and the pre-COVID-19 period in Yousaf et al. (22).^27^ One spillover is from the currency market to the cryptocurrency market. From the above discussion, we find that during the pre-COVID-19 period the stock and gold markets play the role of risk transmitters in this study because the stock market can affect both the oil and cryptocurrency markets and the gold market can affect both the oil and cryptocurrency markets. We also find that during the pre-COVID-19 period the cryptocurrency market plays the role of risk receiver in this study because the stock, oil, gold, and currency markets can affect the cryptocurrency market. On the contrary, we find that during the post-COVID-19 period the oil market plays the role of risk receiver in this study because the stock, gold, and cryptocurrency markets can affect the oil market. Subsequently, we compare the results of volatility spillover for the pre- and post-COVID-19 periods.^28^ We find that, after the COVID-19 pandemic, the significant cases decrease because the significant cases of spillover for the pre-COVID-19 period are seven but four for the post-COVID-19 period. Moreover, we don't find the cases of significant volatility spillover completely disappear after the COVID-19 pandemic. For example, the significant spillover from the stock market to the cryptocurrency market is changed into a slightly significant situation after the COVID-19 pandemic.

Thirdly, from Figure 4, we can observe that irrespectively of the pre-COVID-19 period or the post-COVID-19 period, the correlation within five markets is loosely related as compared with the phenomena found from the volatility spillover. From Figure 4A, we find that during the pre-COVID-19 period there significantly exists a correlation between the stock market and the oil market, which is consistent with the post-SB period in Su (19). We also find that there exist two cases of significant correlation during the post-COVID-19 period as shown in Figure 4B. The two cases of correlation are between the stock market and oil market and between the currency market and cryptocurrency market, which is partially consistent with the post-SB period in Su (19).^29^ Subsequently, we compare the results of correlation for the pre- and post-COVID-19 periods.^30^ We find that, after the COVID-19 pandemic, the significant cases increase because the significant cases of correlation for the pre-COVID-19 period are one but two for the post-COVID-19 period. Moreover, after the COVID-19 pandemic, one significant case of correlation between the currency market and the cryptocurrency market appears.

To sum up, during the pre-COVID-19 period, the volatility spillover (7) is the most significant followed by the return spillover (3) whereas the correlation (1) is the least significant. After the COVID-19 pandemic, the volatility spillover (4) is still the most significant followed by the correlation (2) and the return spillover (2).^31^ In other words, irrespectively of the pre- or post-COVID-19 period, the volatility spillover significantly exists in most of the ten paired markets whereas the return spillover and correlation are significant only for the few paired markets. These results indicate that the factor of short-term risk is more important than the factors of both the short-term return and the long-term correlation for the investors and fund managers. We also find some impact of the COVID-19 pandemic on the interactions. Firstly, after the COVID-19 pandemic, the significant cases for the return spillover and volatility spillover decreased but those for the correlation increased. Secondly, after the COVID-19 pandemic, two return spillover cases completely disappear whereas one correlation case and another return spillover case appear. Hence, from the viewpoint of the variation of the total number of significant cases, the impact of the COVID-19 pandemic on the volatility spillover is greater than the return spillover and the correlation.^32^ However, from the viewpoint of a significant case appearing or disappearing after the COVID-19 pandemic, the impact of the COVID-19 pandemic on the return spillover is the greatest followed by the correlation whereas the volatility spillover is not affected by the COVID-19 pandemic. These results imply that, regarding the short-term return, the investors and fund managers must make different short-term investment strategies for the pre- and post-COVID-19 periods.

### The results of optimal asset allocation in the era of the COVID-19 pandemic

**Figure 5:**
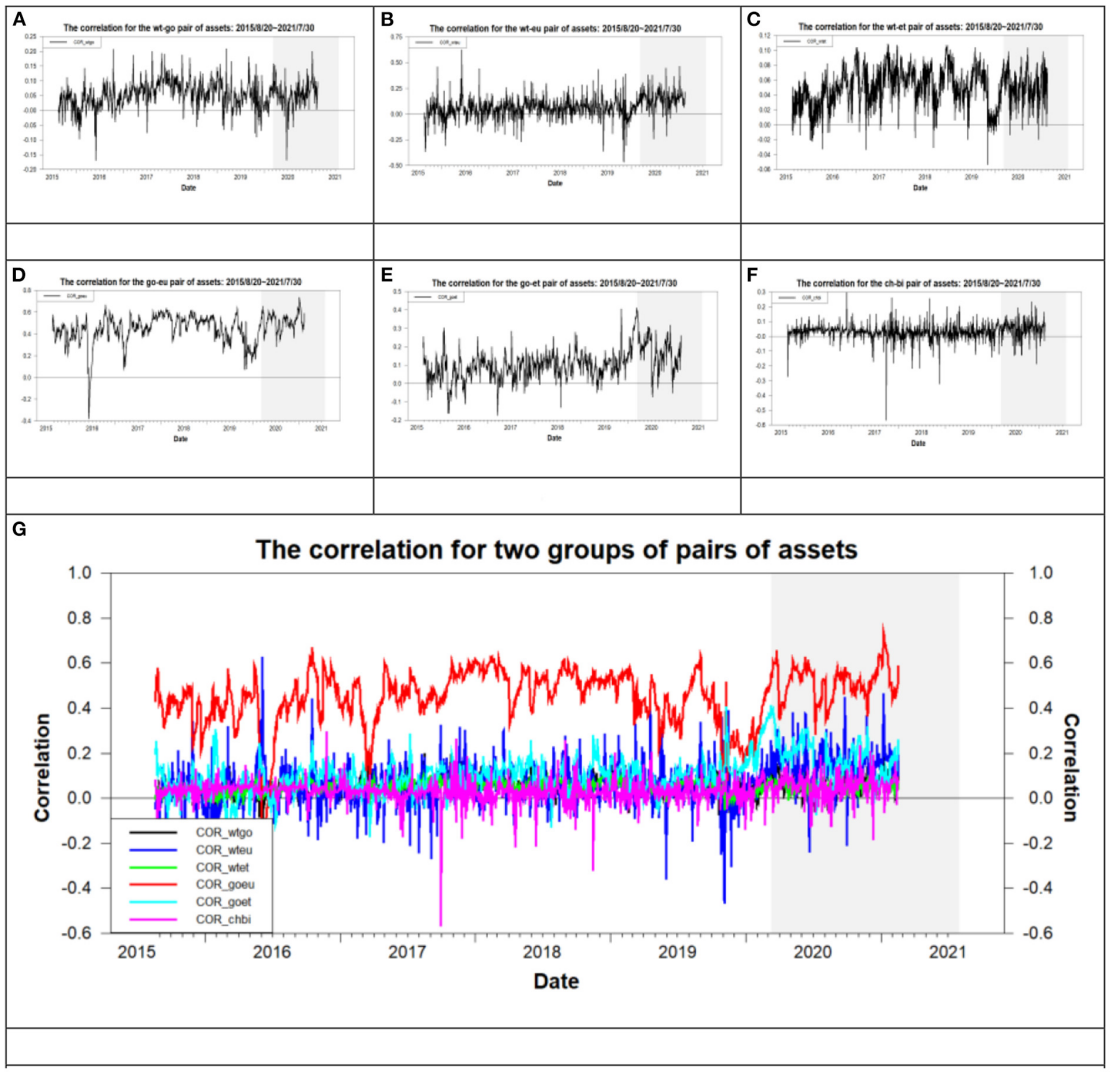
The time-varying correlation for six pairs of assets. **(A)** wt-go, **(B)** wt-eu, **(C)** wt-et, **(D)** go-eu, **(E)** go-et, **(F)** ch-bi, and **(G)** all six pairs of assets.

In this subsection, we explore the issue of optimal asset allocation for the ten paired markets or 23 pairs of assets during the pre- and post-COVID-19 subperiods to examine the variation of performance of asset allocation in the crisis era of the COVID-19 pandemic. The optimal portfolio is the minimum variance portfolio (MVP) obtained from the MDWI approach of Su (41) or Kroner and Ng (42). The performance of asset allocation is measured by the risk-adjusted return. Table 5 lists the results of optimal asset allocation and its corresponding optimal portfolio's performance for the overall period and its two subperiods. From the data listed in the columns “ρ” and “*R*^*a*^” of Table 5, we find the following phenomena when the values of “ρ” (or “*R*^*a*^”) for the overall, and pre- and post-COVID-19 periods are compared at the same pair of assets. First, regarding each pair of assets, the greatest and smallest values of “ρ” (or “*R*^*a*^”) are all dispersed at the pre- or post-COVID-19 period. This result implies that the values of correlation or risk-adjusted return for the overall period are nearly the average values of correlation or risk-adjusted return for the pre- and post-COVID-19 periods. This phenomenon is similar to that found in the mean return, standard deviation, and risk-adjusted return in Table 2. Second, except for the dj-go, wt-go, and wt-et pairs of assets, the greatest values of correlation are all distributed in the post-COVID-19 period. This result indicates that the COVID-19 pandemic crisis increases the relation between assets on the price trend more closely in a long term. Third, except for the go-ch, go-et, and go-li pairs of assets, the greatest values of risk-adjusted return are all distributed in the post-COVID-19 period. This result indicates that the QE implemented after the COVID-19 pandemic crisis increases the risk-adjusted return. These results infer that the investors and fund managers can take a long position during the post-COVID-19 period but they should withdraw capital from the market when the QE tapering is executed. Fourth, regarding the overall period, the values of correlation are between −0.0089 for the dj-go and 0.4507 for the go-eu whereas the values of risk-adjusted return range from 0.0102 for the wt-eu to 0.0883 for the dj-bi. Finally, Figure 5 illustrates the trend of correlation for six pairs of assets during the overall period. The six pairs of assets are the wt-go, wt-eu, wt-et, go-eu, go-et, and ch-bi pairs assets, which are suitable to hedge as described in subsection 5.3. From Figure 5, we find that the correlation of the go-eu pair of assets has the greatest value correlation and it is >0 for most of the study period. As to the other pairs of assets, the correlation fluctuates at zero value during the entire study period.

**Table 5:** The optimal asset allocation and the measure of its risk diversification.

	**dj-wt**			**dj-go**			**dj-ch**		
	ρ	*w*_1_	*R* ^*a*^	ρ	*w*_1_	*R* ^*a*^	ρ	*w*_1_	*R* ^*a*^
Overall	0.2561	0.9524	0.0641	−0.0089	0.5024	0.0823	0.0245	0.9643	0.0695
Pre	*0.2395*	0.9454	*0.0522*	**0.0117**	0.4974	*0.0769*	*0.0239*	0.9701	*0.0608*
Post	**0.3107**	0.9755	**0.1034**	*−0.0776*	0.5189	**0.1003**	**0.0263**	0.9454	**0.0980**
	**dj-eu**			**dj-bi**			**dj-et**		
	ρ	*w*_1_	*R* ^*a*^	ρ	*w*_1_	*R* ^*a*^	ρ	*w*_1_	*R* ^*a*^
Overall	0.0053	0.2533	0.0377	0.0234	0.9511	0.0883	0.0759	0.9862	0.0736
Pre	*−0.0438*	0.3057	*0.0188*	*−0.0118*	0.9532	*0.0809*	*0.0455*	0.9880	*0.0641*
Post	**0.1670**	0.0810	**0.0998**	**0.1393**	0.9441	**0.1127**	**0.1759**	0.9803	**0.1048**
	**dj-li**			**wt-go**			**wt-ch**		
	ρ	*w*_1_	*R* ^*a*^	ρ	*w*_1_	*R* ^*a*^	ρ	*w*_1_	*R* ^*a*^
Overall	0.0358	0.9732	0.0735	0.0528	0.1143	0.0344	0.0063	0.7666	0.0240
Pre	*0.0038*	0.9767	*0.0644*	**0.0544**	0.1044	*0.0324*	*−0.0156*	0.7745	*0.0171*
Post	**0.1412**	0.9618	**0.1032**	*0.0473*	0.1469	**0.0406**	**0.0785**	0.7407	**0.0469**
	**wt-eu**			**wt-bi**			**wt-et**		
	ρ	*w*_1_	*R* ^*a*^	ρ	*w*_1_	*R* ^*a*^	ρ	*w*_1_	*R* ^*a*^
Overall	0.0678	0.0276	0.0102	0.0508	0.7229	0.0497	0.0498	0.8636	0.0351
Pre	*0.0521*	0.0343	*−0.0022*	*0.0422*	0.7341	*0.0399*	**0.0516**	0.8990	*0.0186*
Post	**0.1192**	0.0058	**0.0512**	**0.0792**	0.6860	**0.0819**	*0.0439*	0.7473	**0.0891**
	**wt-li**			**go-ch**			**go-eu**		
	ρ	*w*_1_	*R* ^*a*^	ρ	*w*_1_	*R* ^*a*^	ρ	*w*_1_	*R* ^*a*^
Overall	0.0486	0.8230	0.0238	0.0035	0.9597	0.0479	0.4507	0.0667	0.0168
Pre	*0.0436*	0.8391	*0.0130*	*−0.0029*	0.9595	**0.0553**	*0.4486*	0.1025	*0.0083*
Post	**0.0651**	0.7701	**0.0595**	**0.0248**	0.9603	*0.0238*	**0.4576**	−0.0508	**0.0448**
	**go-et**			**go-li**			**ch-bi**		
	ρ	*w*_1_	*R* ^*a*^	ρ	*w*_1_	*R* ^*a*^	ρ	*w*_1_	*R* ^*a*^
Overall	0.1047	0.9959	0.0421	0.0538	0.9730	0.0470	0.0315	0.4274	0.0868
Pre	*0.0822*	0.9953	**0.0448**	*0.0293*	0.9686	**0.0524**	*0.0265*	0.4257	*0.0816*
Post	**0.1786**	0.9979	*0.0332*	**0.1341**	0.9875	*0.0294*	**0.0477**	0.4329	**0.1039**
	**ch-et**			**ch-li**			**eu-bi**		
	ρ	*w*_1_	*R* ^*a*^	ρ	*w*_1_	*R* ^*a*^	ρ	*w*_1_	*R* ^*a*^
Overall	0.0112	0.6165	0.0689	0.0279	0.5783	0.0491	0.0791	0.9883	0.0171
Pre	*−0.0181*	0.6334	*0.0619*	*0.0131*	0.5838	*0.0462*	*0.0229*	0.9791	*0.0090*
Post	**0.1077**	0.5608	**0.0919**	**0.0765**	0.5603	**0.0585**	**0.2636**	1.0185	**0.0438**
	**eu-et**			**eu-li**					
	ρ	*w*_1_	*R* ^*a*^	ρ	*w*_1_	*R* ^*a*^			
Overall	0.0694	1.0002	0.0110	0.0370	0.9951	0.0118			
Pre	*0.0278*	0.9972	*−0.0013*	*−0.0245*	0.9898	*0.0022*			
Post	**0.2060**	1.0099	**0.0515**	**0.2392**	1.0127	**0.0433**			

1. “dj” denotes the Dow Jones Industrial Average index in the stock market; “wt” and “go” represent the WTI crude oil and gold in the commodity market, respectively; “ch” and “eu” denote the Chinese yuan (CNY) and Euro in the currency market, respectively, and “bi,” “et,” and “li” denote the abbreviations of Bitcoin, Ethereum, and Litecoin in the cryptocurrency market, respectively. 2. ρ denotes the average value of correlation for a pair of assets during a specific period. *w*_1_ is the average weight forecast of the first component asset of the minimum variance portfolio (MVP) based on the MDWI approach of Su (41) or Kroner and Ng (42). 3. *R*^*a*^ denotes the realized risk-adjusted returns of the MVP, which is obtained by the return of the MVP divided by the standard deviation of the MVP. 4. The bold font in columns “ρ” (or “*R*^*a*^”) for a specific pair of assets denotes the largest value when the values of “ρ” (or “*R*^*a*^”) for three periods are compared with each other. 5. The italic font in columns “ρ” (or “*R*^*a*^”) for a specific pair of assets denotes the smallest value when the values of “ρ” (or “*R*^*a*^”) for three periods are compared with each other.

**Figure 6:**
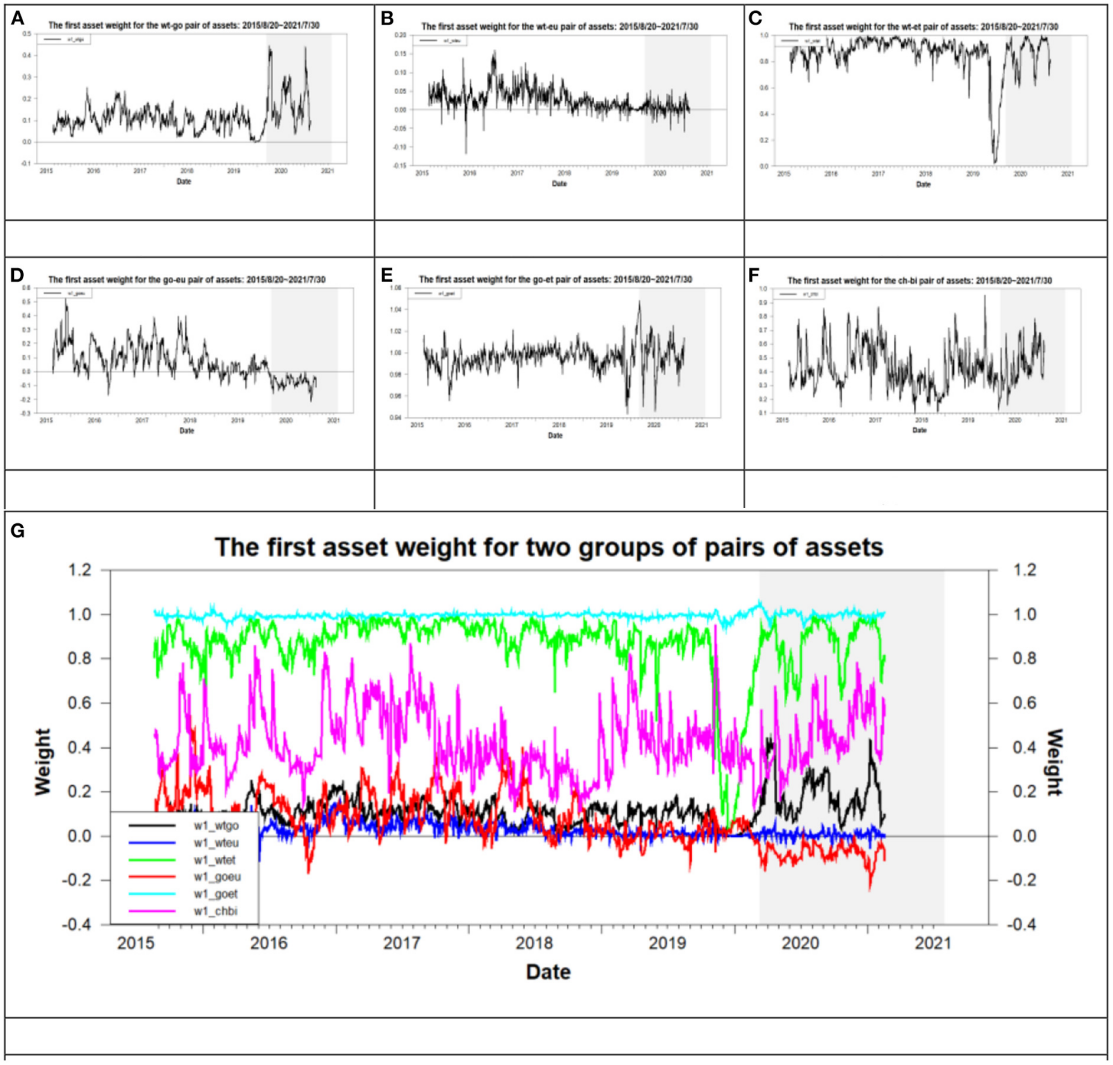
The time-varying weight for six pairs of assets. **(A)** wt-go, **(B)** wt-eu, **(C)** wt-et, **(D)** go-eu, **(E)** go-et, **(F)** ch-bi, and **(G)** all six pairs of assets.

From the data listed in the column “*w*_1_” of Table 5, we probably know, according to the weight of the first component asset on the minimum variance portfolio, the sequence of values of the weight of eight assets from the largest to the smallest. For example, the value of “*w*_1_” of the dj-wt pair of assets is 0.9524 during the overall period. That is, the weight of Dow Jones is 0.9524, which is >0.5. This indicates that the weight of Dow Jones is greater than that of WTI for the MVP of the dj-wt pair of assets. The above result is recorded as “dj-wt (dj > wt)”. With the same inference process, we can record the results for the remaining pairs of assets as dj-go (dj > go), dj-ch (dj > ch), dj-eu (eu > dj), dj-bi (dj > bi), dj-et (dj > et), dj-li (dj > li), wt-go (go > wt), wt-ch (wt > ch), wt-eu (eu > wt), wt-bi (wt > bi), wt-et (wt > et), wt-li(wt > li), go-ch (go > ch), go-eu (eu > go), go-et (go > et), go-li (go > li), ch-bi (bi > ch), ch-et (ch > et), ch-li (ch > li), eu-bi (eu > bi), eu-et (eu > et), eu-li (eu > li). The inequality in the bracket beside a pair of assets represents the weight comparison result of assets for a pair of assets. According to the above inequalities eu > dj of the dj-eu, dj > go of dj-go, go > wt of the wt-go, wt > bi of the wt-bi, bi > ch of the ch-bi, ch > et of the ch-et, and ch > li of the ch-li, we probably infer the sequence of values of the weight of eight assets from the largest to the smallest as “eu > dj > go > wt > bi > ch > et, or li”. Based on the above weight sequence of assets, the euro is the most important asset whereas Ethereum or Litecoin is the least important asset when the investors construct an optimal portfolio based on the minimum risk. This indicates that investors and fund managers should select Euro to construct a portfolio to achieve risk diversification.^33^ In addition, Figure 6 illustrates the trend of the weight of the first component asset of the minimum variance portfolio for six pairs of assets mentioned above during the overall period. As shown by the weight scale of the vertical axis in Figure 6, we find that the weight of the first component asset for the go-et pair of assets has the greatest value followed by that for the wt-et and ch-bi during the overall period. This result indicates that the weight of gold is greater than WTI. Moreover, the weight of WTI is greater than CNY. These results are the same as those found above.

### The results of an optimal hedged strategy in the era of the COVID-19 pandemic

In this subsection, the issue of an optimal hedged strategy for the ten paired markets or 23 pairs of assets during the pre- and post-COVID-19 subperiods is explored to examine the variations of performance of the hedged strategy in the era of the COVID-19 pandemic. The optimal hedged portfolio is constructed by minimizing the risk of this hedged portfolio, which is proposed by Kroner and Sultan (44). The performance of the hedged strategy is measured by the hedge ratios proposed by Kroner and Sultan (44) and the hedging effectiveness proposed by Ku et al. (45). The lower value of hedge ratio represents the lower hedging cost. A higher value of hedge effectiveness indicates the greater hedge effectiveness in terms of the portfolio's variance reduction. Notably, the lower value of hedging cost and the greater value of hedge effectiveness denote the better performance of the hedged portfolio.

Table 6 lists the results of two optimal hedged strategies and the performance of their corresponding hedged portfolios for the overall period and its two subperiods. From the data listed in columns “β_1_,” “β_2_,” “$HE_{1}^{h}$,” and “$HE_{2}^{h}$” in Table 6, the values of hedging effectiveness (HE) and the hedge ratio are negative except for very few pairs of assets. This indicates that most of the pairs of the data are not suitable to hedge each other. The reasons are illustrated as follows. First of all, the negative value of hedging effectiveness (HE) indicates that the risk of the hedged portfolio increases after being hedged as shown in equation (16). Secondly, the negative values of covariance or correlation will produce the negative values of the hedge ratio as shown in equation (15). Therefore, taking a short position should be changed into taking a long position. These phenomena also appeared in the hedge ratio for the bond-stock and bond-oil pairs of assets in Dutta et al. (20) and gold and the G7 stock indices in Shahzad et al. (48). The aforementioned two phenomena violate the principle of a hedge. Hence, regarding the few pairs of assets, which are suitable to hedge, they have positive values on both the hedge ratio and hedging effectiveness. They can be divided into the following three groups. The first group includes the dj-wt, dj-ch, and dj-et pairs of assets. The dj-wt and dj-et pairs of assets are only suitable to construct the second hedged portfolio; that is, taking a short position of Dow Jones and a long position of WTI and Ethereum, respectively. The reason is that the values of “β_2_” and “$HE_{2}^{h}$” for the two pairs of assets are all positive for the overall period and two subperiods. At the same inference process, the dj-ch pair of assets is only suitable to establish the first hedged portfolio; that is, taking a long position of Dow Jones and a short position of Chinese yuan.^34^ The reason is that the values of “β_1_” and “$HE_{1}^{h}$” for the dj-ch pair of assets are all positive for the overall period and two subperiods. The second group contains the wt-go, wt-eu, and wt-et pairs of assets. The three pairs of assets are only suitable to construct the first hedged portfolio because the values of “β_1_” and “$HE_{1}^{h}$” for the pairs of assets in this group are all positive for the overall period and two subperiods. That is, taking a long position of WTI is accompanied with a short position of gold, Euro, or Ethereum, respectively. The third group includes the go-eu, go-et, and ch-bi pairs of assets. The three pairs of assets are suitable to construct the first and second hedged portfolios. The reason is that the values of “β_1_,” “β_2_,” “$HE_{1}^{h}$,” and “$HE_{2}^{h}$” for the pairs of assets in this group are all positive for the overall period and two subperiods.

**Table 6:** The optimal hedge ratio and its risk reduction effectiveness.

		**dj-wt**					**dj-go**			
	**ρ**	**β_1_**	**β_2_**	** $HE_{1}^{h}$ **	** $HE_{2}^{h}$ **	**ρ**	**β_1_**	**β_2_**	** $HE_{1}^{h}$ **	** $HE_{2}^{h}$ **
Overall	0.2561	0.0938	0.7612	0.0351	0.0567	−0.0089	×	×	×	×
Pre	0.2395	0.0856	**0.7386**	0.0469	*0.0448*	0.0117	0.0123	0.0124	×	×
Post	0.3107	0.1205	*0.8355*	×	0.0956	−0.0776	×	×	0.0015	0.0012
		**dj-ch**					**dj-eu**			
	ρ	β_1_	β_2_	$HE_{1}^{h}$	$HE_{2}^{h}$	ρ	β_1_	β_2_	$HE_{1}^{h}$	$HE_{2}^{h}$
Overall	0.0245	0.0038	0.1743	*0.0004*	×	0.0053	0.0483	×	×	×
Pre	0.0239	**0.0036**	0.1781	*0.0004*	0.0001	−0.0438	×	×	×	0.0009
Post	0.0263	*0.0048*	0.1620	**0.0007**	×	0.1670	0.4624	0.0691	0.0063	−0.0077
		**dj-bi**					**dj-et**			
	ρ	β_1_	β_2_	$HE_{1}^{h}$	$HE_{2}^{h}$	ρ	β_1_	β_2_	$HE_{1}^{h}$	$HE_{2}^{h}$
Overall	0.0234	0.0090	0.0731	×	0.0009	0.0759	0.0148	0.5833	×	0.0074
Pre	−0.0118	×	×	×	×	0.0455	0.0054	0.4653	×	*0.0003*
Post	0.1393	0.0435	0.6046	0.0084	0.0058	0.1759	0.0456	*0.9707*	0.0161	**0.0307**
		**dj-li**					**wt-go**			
	ρ	β_1_	β_2_	$HE_{1}^{h}$	$HE_{2}^{h}$	ρ	β_1_	β_2_	$HE_{1}^{h}$	$HE_{2}^{h}$
Overall	0.0358	0.0085	0.3311	×	0.0015	0.0528	0.1491	0.0210	0.0028	×
Pre	0.0038	0.0005	0.1364	×	×	0.0544	*0.1508*	0.0205	0.0030	0.0021
Post	0.1412	0.0347	0.9703	0.0114	0.0144	0.0473	**0.1435**	0.0227	*0.0021*	×
		**wt-ch**					**wt-eu**			
	ρ	β_1_	β_2_	$HE_{1}^{h}$	$HE_{2}^{h}$	ρ	β_1_	β_2_	$HE_{1}^{h}$	$HE_{2}^{h}$
Overall	0.0063	×	0.0257	×	×	0.0678	0.3461	0.0141	0.0045	×
Pre	−0.0156	×	×	0.0000	×	0.0521	**0.2440**	0.0116	*0.0023*	0.0003
Post	0.0785	0.0246	0.2132	×	0.0002	0.1192	*0.6813*	0.0222	0.0120	×
		**wt-bi**					**wt-et**			
	ρ	β_1_	β_2_	$HE_{1}^{h}$	$HE_{2}^{h}$	ρ	β_1_	β_2_	$HE_{1}^{h}$	$HE_{2}^{h}$
Overall	0.0508	0.0407	0.0840	×	×	0.0498	0.0180	0.1611	0.0019	×
Pre	0.0422	0.0301	0.0762	×	0.0005	0.0516	0.0174	0.1697	**0.0021**	0.0021
Post	0.0792	0.0753	0.1095	×	×	0.0439	0.0200	0.1329	*0.0015*	×
		**wt-li**					**go-ch**			
	ρ	β_1_	β_2_	$HE_{1}^{h}$	$HE_{2}^{h}$	ρ	β_1_	β_2_	$HE_{1}^{h}$	$HE_{2}^{h}$
Overall	0.0486	0.0215	0.1496	0.0009	×	0.0035	0.0004	0.0433	0.0000	×
Pre	0.0436	0.0194	0.1289	×	×	−0.0029	×	0.0171	0.0000	×
Post	0.0651	0.0285	0.2177	0.0042	×	0.0248	0.0050	0.1292	0.0001	0.0003
		**go-eu**					**go-et**			
	ρ	β_1_	β_2_	$HE_{1}^{h}$	$HE_{2}^{h}$	ρ	β_1_	β_2_	$HE_{1}^{h}$	$HE_{2}^{h}$
Overall	0.4507	0.9078	0.2424	0.1869	0.1759	0.1047	0.0153	0.8052	0.0058	0.0120
Pre	0.4486	**0.7844**	*0.2666*	*0.1857*	0.1895	0.0822	0.0097	**0.7452**	*0.0008*	*0.0063*
Post	0.4576	*1.3130*	0.1629	0.1908	*0.1312*	0.1786	*0.0336*	*1.0020*	**0.0221**	0.0307
		**go-li**					**ch-bi**			
	ρ	β_1_	β_2_	$HE_{1}^{h}$	$HE_{2}^{h}$	ρ	β_1_	β_2_	$HE_{1}^{h}$	$HE_{2}^{h}$
Overall	0.0538	0.0100	0.3359	×	0.0009	0.0315	0.0375	0.0284	0.0008	0.0008
Pre	0.0293	0.0048	0.2162	×	×	0.0265	**0.0313**	0.0242	*0.0005*	*0.0004*
Post	0.1341	0.0269	0.7290	0.0108	0.0136	0.0477	*0.0577*	*0.0421*	**0.0018**	0.0020
		**ch-et**					**ch-li**			
	ρ	β_1_	β_2_	$HE_{1}^{h}$	$HE_{2}^{h}$	ρ	β_1_	β_2_	$HE_{1}^{h}$	$HE_{2}^{h}$
Overall	0.0112	0.0142	0.0049	×	0.0001	0.0279	0.0231	0.0357	0.0002	0.0005
Pre	−0.0181	×	×	×	×	0.0131	0.0090	0.0185	×	×
Post	0.1077	0.1059	0.1223	0.0031	0.0010	0.0765	0.0694	0.0921	0.0033	0.0037
		**eu-bi**					**eu-et**			
	ρ	β_1_	β_2_	$HE_{1}^{h}$	$HE_{2}^{h}$	ρ	β_1_	β_2_	$HE_{1}^{h}$	$HE_{2}^{h}$
Overall	0.0791	0.0094	0.8073	0.0014	×	0.0694	0.0048	1.0469	0.0037	0.0050
Pre	0.0229	0.0040	0.1961	×	×	0.0278	0.0018	0.4296	×	×
Post	0.2636	0.0273	2.8144	0.0374	0.0376	0.2060	0.0145	3.0737	0.0231	0.0251
		**eu-li**								
	ρ	β_1_	β_2_	$HE_{1}^{h}$	$HE_{2}^{h}$					
Overall	0.0370	0.0026	0.5451	0.0010	0.0005					
Pre	−0.0245	×	×	×	×					
Post	0.2392	0.0182	3.5098	0.0169	0.0180					

1. “dj” denotes the Dow Jones Industrial Average index in the stock market; “wt” and “go” respectively represent the WTI crude oil and gold in the commodity market; “ch” and “eu” denote the Chinese yuan (CNY) and Euro in the currency market, respectively; and “bi,” “et,” and “li” denote the abbreviation of Bitcoin, Ethereum, and Litecoin in the cryptocurrency market, respectively. 2. For a pair of assets, β_1_ denotes the average value of the hedge ratio by buying the first asset and selling the second asset whereas β_2_ represents the average value of the hedge ratio by buying the second asset and selling the first asset. On the other hand, $HE_{1}^{h}$ denotes the average value of risk reduction effectiveness of a hedged portfolio by buying the first asset and selling the second asset whereas $HE_{2}^{h}$ denotes the average value of risk reduction effectiveness of hedged portfolio by buying the second asset and selling the first asset. 3. The symbol “ × ” in columns “$HE_{1}^{h}$” and “$HE_{2}^{h}$” denotes the value of hedging effectiveness (HE) is negative. This indicates that the risk of a hedged portfolio increases being after hedged. Thus, this pair of assets is not suitable to hedge each other. 4. The symbol “ × ” in columns “β_1_” and “β_2_” denotes the value of the hedge ratio is negative. This indicates that the value of covariance or correlation is negative. Then, it should do a short position is changed to do a long position, indicating that it violates the principle of the hedge. Thus, this pair of assets is not suitable to hedge each other. 5. The bold font in columns “β_1_” and “β_2_” (“$HE_{1}^{h}$” and “$HE_{2}^{h}$”) denotes the smallest (largest) value when the values of “β_1_” and “β_2_” (“$HE_{1}^{h}$” and “$HE_{2}^{h}$”) for a specific pair of assets at the same panel (representing the overall period and its two subperiods) are compared each other. The above comparison is executed based on the symbol “ × ” does not appear in “β_*i*_” or “$HE_{i}^{h}$” where *i* = 1 *or* 2. 6. The italic font in columns “β_1_” and “β_2_” (“$HE_{1}^{h}$” and “$HE_{2}^{h}$”) denotes the largest (smallest) value when the values of “β_1_” and “β_2_” (“$HE_{1}^{h}$” and “$HE_{2}^{h}$”) for a specific pair of assets at the same panel (representing the overall period and its two subperiods) are compared each other. The above comparison is executed based on the symbol “ × ” does not appear in “β_*i*_” or “$HE_{i}^{h}$” where *i* = 1 *or* 2. 7. The shade font in the column “β_2_” for the dj-wt and dj-et pairs of assets denotes the smaller value of “β_2_” when the values of “β_2_” for the dj-wt and dj-et pairs of assets are compared each other based on the same subperiod. Conversely, the shaded font in the column “$HE_{2}^{h}$” for the dj-wt and dj-et pairs of assets denotes the greater value of “$HE_{2}^{h}$” when the values of “$HE_{2}^{h}$” for the dj-wt and dj-et pairs of assets are compared each other based on the same subperiod. 8. The shade font in the column “β_1_” (“$HE_{1}^{h}$”) for the wt-go, wt-eu, and wt-et pairs of assets denotes the smallest (greatest) value of “β_1_” (“$HE_{1}^{h}$”) when the values of “β_1_” (“$HE_{1}^{h}$”) for the above three pairs of assets are compared each other based on the same subperiod. 9. The shade font in the column “β_1_” (or “β_2_”) for the go-eu, go-et, and ch-bi pairs of assets denotes the smaller value of “β_1_” (or “β_2_”) when the values of “β_1_” and “β_2_” are compared each other based on the same subperiod and the same pair of assets. Conversely, the shaded font in the column “$HE_{1}^{h}$” (or “$HE_{2}^{h}$”) for the go-eu, go-et, and ch-bi pairs of assets denote the greater value of “$HE_{1}^{h}$” (or “$HE_{2}^{h}$”) when the values of “$HE_{1}^{h}$” and “$HE_{2}^{h}$” are compared each other based on the same subperiod and the same pair of assets.

From the data in these three groups, the following phenomena simultaneously appear in the pair of assets in the three groups. First of all, the greatest and smallest values of the hedge ratio always appear in the pre- or post-COVID-19 period when the values of the hedge ratio for the overall period, and the pre- and post-COVID-19 periods are compared with each other at the same pair of assets. The same results also occur in the hedging effectiveness. This indicates that the values of the hedge ratio (or hedging effectiveness) for the overall period are nearly the average values of the hedge ratio (or hedging effectiveness) for the pre- and post-COVID-19 periods. This phenomenon is similar to that found in the mean return, standard deviation, and risk-adjusted return in Table 2. Secondly, when the values of the hedge ratio (or hedging effectiveness) for the pre- and post-COVID-19 periods are compared with each other, if the lower values of the hedge ratio appear in the pre-COVID-19 period, then the higher values of hedging effectiveness appear in the post-COVID-19 period, and vice versa. This indicates that the optimal hedge strategy cannot be selected simultaneously based on the hedge cost and hedging effectiveness. That is, based on the criterion of lower hedge cost, if the first asset is bought and the second asset is sold to hedge, then this strategy is violated based on the criterion of higher hedging effectiveness. For example, except for the wt-et, the higher (lower) values of the hedge ratio and the greater (lower) value of hedge effectiveness occur in the post-COVID-19 period simultaneously. This indicates that investors need to spend more (few) hedge costs to get more (less) risk reduction during the post-COVID-19 period. Regarding the exception of the wt-et pair of assets, the investors can spend a few hedge costs to get more risk reduction during the pre-COVID-19 period.

Regarding the pairs of assets in the first group such as the dj-wt and dj-et, if the investors take a short position of Dow Jones, then they can long position of WTI or Ethereum to do the hedge.^35^ Subsequently, I compare the values of “β_2_” (or “$HE_{2}^{h}$”) for the dj-wt and dj-et pairs of assets based on the same subperiod to explore which asset (WTI or Ethereum) is the best hedging asset of the Dow Jones. We find that the smaller value of “β_2_” appears in the dj-et and dj-wt during the pre- and post-COVID-19 periods, respectively. Conversely, the greater value of “$HE_{2}^{h}$” appears in the dj-wt during the pre- and post-COVID-19 periods. This result indicates that Ethereum and WTI are the optimal hedge assets of Dow Jones, respectively, during the pre- and post-COVID-19 periods from the perspective of hedge cost. Moreover, from the perspective of risk reduction, WTI is the optimal hedge asset of Dow Jones during the pre- and post-COVID-19 periods. Hence, WTI is the optimal hedge asset of Dow Jones during the post-COVID-19 period from the perspectives of hedge cost and risk reduction.

**Figure 7:**
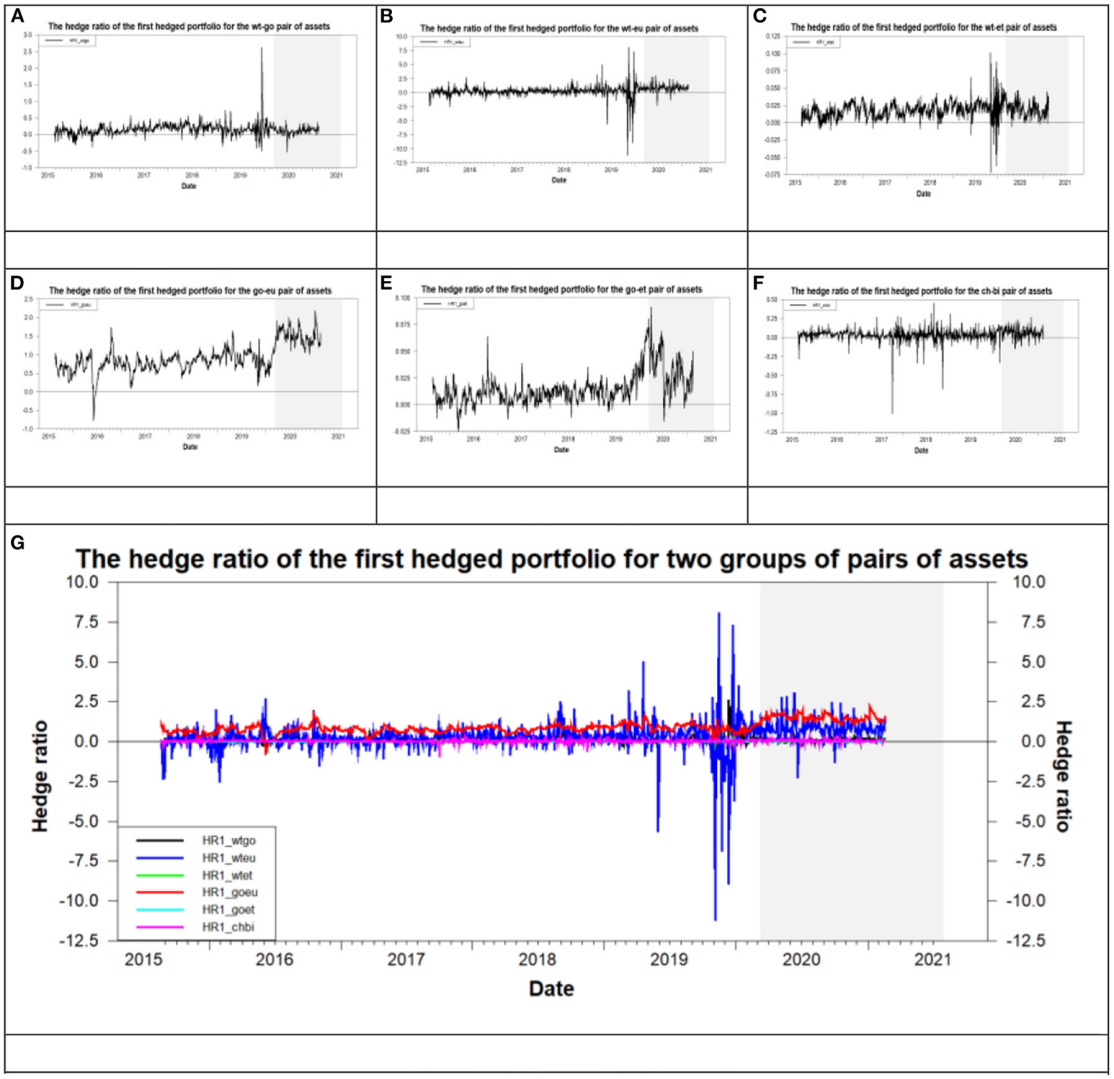
The time-varying hedge ratio for the six pairs of assets. **(A)** wt-go, **(B)** wt-eu, **(C)** wt-et, **(D)** go-eu, **(E)** go-et, **(F)** ch-bi, and **(G)** all six pairs of assets.

**Figure 8:**
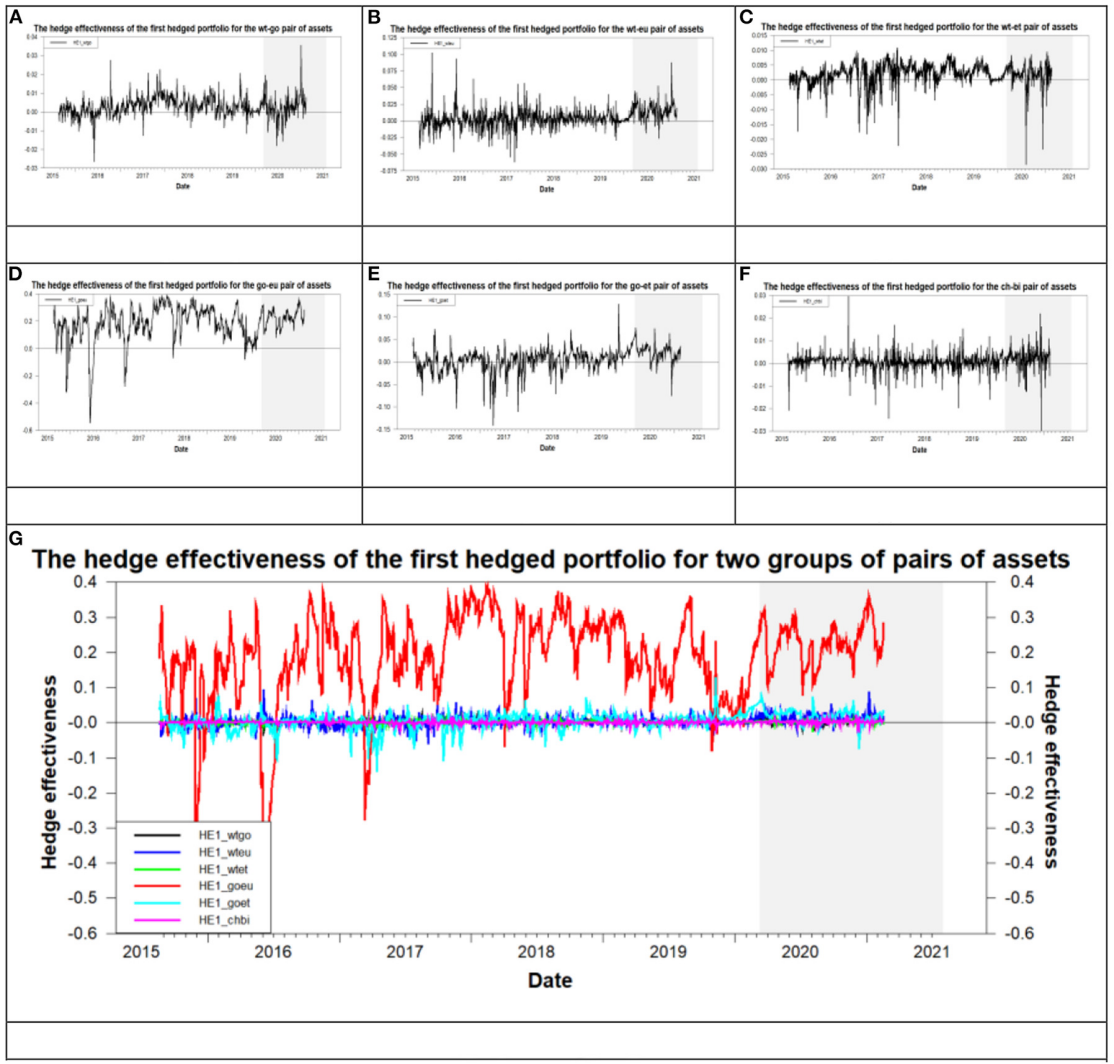
The time-varying hedge effectiveness for six pairs of assets. **(A)** wt-go, **(B)** wt-eu, **(C)** wt-et, **(D)** go-eu, **(E)** go-et, **(F)** ch-bi, and **(G)** all six pairs of assets.

Regarding the pairs of assets in the second group such as the wt-go, wt-eu, and wt-et, if the investors bear the WTI asset, then they can sell the gold, Euro, or Ethereum to do the hedge. Subsequently, I compare the values of “β_1_” (or “$HE_{1}^{h}$”) for the wt-go, wt-eu, and wt-et pairs of assets based on the same subperiod to explore which asset (gold, euro, or Ethereum) is the best hedging asset of the WTI. We find that the smallest value of “β_1_” appears at the wt-et during the pre- and post-COVID-19 periods. On the other hand, the greatest value of “$HE_{1}^{h}$” appears at the wt-go and wt-eu during the pre- and post-COVID-19 periods, respectively. From Figures 7, 8, we also find the above phenomena.^36^ Figures 7, 8 illustrates the trend of the hedge ratio and hedge effectiveness for the pairs of assets in the second and third groups during the overall period, respectively. This result indicates that Ethereum is the optimal hedge asset of WTI during the pre- and post-COVID-19 periods from the perspective of hedge cost. Moreover, from the perspective of risk reduction, gold and Euro are the optimal hedge assets of WTI during the pre- and post-COVID-19 periods, respectively. In addition, we also find that hedge ratio and hedge effectiveness are not stable over time as shown by the time-variation in the hedge ratio and hedge effectiveness in Figures 7, 8. This phenomenon is in line with Dutta et al. (20) and Shahzad et al. (49).

Regarding the pairs of assets in the third group such as the go-eu, go-et, and ch-bi pairs of assets, they are suitable to construct the first and second hedge portfolios. Subsequently, based on the same subperiod, we compare the values of “β_1_” and “β_2_” (or “$HE_{1}^{h}$” and “$HE_{2}^{h}$”) for each of the go-eu, go-et, and ch-bi pairs of assets to investigate which strategy, the first or second hedged portfolio, is better. We find the following results by using the same inference process of the pairs of assets in the first and second groups. First, irrespectively of the pre- or post-COVID-19 period, the first hedge strategy is suitable for the go-et whereas the second hedge strategy is suitable for the go-eu and ch-bi from the perspective of hedge cost. Second, from the perspective of risk reduction, we can't get the consistent results found from the perspective of hedge cost. That is, the optimal hedge strategy varies with the pre- and post-COVID-19 periods except for the go-et. Regarding the exception of go-et, the second hedge strategy is suitable for the pre- and post-COVID-19 periods. Third, from the perspectives of both hedge cost and risk reduction, the second hedge strategy is suitable for the go-eu and ch-bi pairs of assets during the pre- and post-COVID-19 periods, respectively. Finally, we compare the values of “β_1_” (or “$HE_{1}^{h}$”) for the go-eu and go-et pairs of assets based on the same subperiod to explore which asset (euro or Ethereum) is the best hedging asset of gold. We find that Ethereum is the optimal hedge asset of gold during the pre- and post-COVID-19 periods from the perspective of hedge cost. Because the value of “β_1_” for the go-et is smaller than that for the go-eu at the pre- and post-COVID-19 periods. This result is also found in Figures 7D,E.^37^ Conversely, from the perspective of risk reduction, the Euro is the optimal hedge asset of gold during the pre- and post-COVID-19 periods. Because the value of “$HE_{1}^{h}$” for the go-eu is greater than that for the go-et at the pre- and post-COVID-19 periods. This result is also found in Figures 8D,E.^38^ From the above discussion, regarding these few pairs of assets, the optimal hedge asset with the fewer hedge cost is accompanied by less risk reduction and vice versa. This indicates that investors and fund managers must encounter the trade-off between hedge cost and risk reduction.

## Conclusion

This study utilizes a bivariate BEKK-GARCH model with two time-dummy variables to investigate how the COVID-19 pandemic crisis affects the interactions between the stock, oil, gold, currency, and cryptocurrency markets. The impact of the COVID-19 pandemic crisis on the optimal asset allocation and optimal hedged strategy is also discussed.

The empirical findings can be summarized as follows. Firstly, irrespectively of the pre- or post-COVID-19 period, the volatility spillover significantly exists in most of the ten paired markets whereas the return spillover and correlation are significant only for the few paired markets. Secondly, from the viewpoint of the significant case appearing or disappearing after the COVID-19 pandemic, the impact of the COVID-19 pandemic on the return spillover is the greatest followed by the correlation whereas the volatility spillover is not affected by the COVID-19 pandemic. Thirdly, the QE implemented after the COVID-19 pandemic crisis increases the risk-adjusted return for each asset and each minimum variance portfolio (MVP) and raises the correlation between the two assets. Fourthly, except for a few pairs of assets, most of the pairs of assets are not suitable to hedge each other because of the following two reasons. First, the risk of a hedged portfolio increases after being hedged. Second, when the investors own a long position, they should take a short position to hedge but are changed into taking a long position. Regarding these few pairs of assets, the optimal hedge asset with the fewer hedge cost is accompanied by less risk reduction and vice versa. Finally, in eight assets Euro owns the lowest return and the smallest risk whereas Ethereum bears the greatest return and the highest risk. Moreover, within eight assets Euro and Ethereum, respectively, take the most and least capital weight in constructing an optimal portfolio. In addition, when hedging WTI or gold, Euro can get the greatest risk reduction whereas Ethereum can spend the cheapest hedge cost.^39^

Based on the above empirical results, I propose the following important policy implications for the investors and fund managers. Firstly, the investors and fund managers can use the volatility of one market to predict the volatility of another market during the entire study period. For example, they can use the volatility of the stock, gold, and cryptocurrency markets to predict the volatility of the oil market. Secondly, the investors and fund managers can take a long position during the post-COVID-19 period but they should withdraw capital from the market when the QE tapering is executed. Thirdly, the investors and fund managers should choose Euro as the main component asset to construct a portfolio to achieve risk diversification. Finally, when hedging gold or WTI the investors and fund managers, who care about the hedge cost more, should select Ethereum to hedge but Euro for the investors and fund managers, who care about the risk reduction more.

Even though this study has provided a comprehensive analysis of the interaction between the stock, oil, gold, currency, and cryptocurrency markets during the period including the COVID-19 pandemic crisis, the results still need a robust check for another crisis and another empirical model. The global financial crisis, a similar crisis, also implemented the QE policy during the crisis period. Another bivariate GARCH model, the DCC-GARCH model, was popularly used to explore the interaction issue in the past literature. Thus, regarding the issues investigated in this study, I will examine the difference between the COVID-19 pandemic crisis and the global financial crisis, and the dissimilarity between the bivariate BEKK-GARCH model and DCC-GARCH model in future research.

## Data availability statement

The original contributions presented in the study are included in the article/supplementary material, further inquiries can be directed to the corresponding author/s.

## Author contributions

J-BS: conceptualization, methodology, software, validation, formal analysis, investigation, writing—original draft preparation, and visualization. Y-SK: resources, data curation, writing—review and editing, project administration, and funding acquisition. All authors contributed to the article and approved the submitted version.

## Conflict of interest

The authors declare that the research was conducted in the absence of any commercial or financial relationships that could be construed as a potential conflict of interest.

## Publisher's note

All claims expressed in this article are solely those of the authors and do not necessarily represent those of their affiliated organizations, or those of the publisher, the editors and the reviewers. Any product that may be evaluated in this article, or claim that may be made by its manufacturer, is not guaranteed or endorsed by the publisher.

## Footnotes

^1^The coronavirus disease 2019 (COVID-19) was first identified in the Chinese city of Wuhan in December 2019 and it quickly spread to other parts of mainland China and even around the world. Then, the World Health Organization (WHO) declared a pandemic on March 11, 2020.

^2^The detailed reasons are that oil is an energy source and it is a factor of production for an enterprise and factory. Therefore, the factory must pay the currency to buy the factors of production for an enterprise and factory to produce its product. Subsequently, an enterprise or factory also receives currency when they sell their products to earn a profit. Notably, the stock is a representation of an enterprise's value which is measured by the profit obtained from selling its products. In addition, gold is a good hedged product for an enterprise when an enterprise works to keep its value not being violent.

^3^See, the website “https://www.sofi.com/learn/content/benefits-of-crypto/” for more details.

^4^The Dow Jones index and WTI crude oil are used to represent the stock market and oil market, respectively. The CNY and Euro are used to represent the currency market whereas Bitcoin, Ethereum, and Litecoin are used to represent the cryptocurrency market.

^5^The interactions between markets in this study include the correlation in the long-term and the return and volatility spillovers in the short-term.

^6^The 23 pairs of assets include dj-wt, dj-go, dj-ch, dj-eu, dj-bi, dj-et, dj-li, wt-go, wt-ch, wt-eu, wt-bi, wt-et, wt-li, go-ch, go-eu, go-et, go-li, ch-bi, ch-et, ch-li, eu-bi, eu-et, and eu-li pairs of assets. The symbols “dj,” “wt,” “go,” “ch,” “eu,” “bi,” “et,” and “li” denote the Dow Jones index, WTI crude oil, gold, Chinese yuan (CNY), Euro, Bitcoin, Ethereum, and Litecoin, respectively. Conversely, 10 paired markets are the stock-oil, stock-gold, stock-currency, stock-cryptocurrency, oil-gold, oil-currency, oil-cryptocurrency, gold-currency, gold-cryptocurrency, and currency-cryptocurrency paired markets.

^7^An optimal portfolio with minimum risk is the minimum variance portfolio (MVP), which is illustrated in Section The optimal asset allocation and the optimal hedged strategy.

^8^Even if the oil and gold belong to the commodity market, this study considers the oil and gold as two different assets because these two assets own different attributes in the commodity market. In addition, oil and gold are the assets in the energy market and metal market, respectively. The ten paired markets are constructed by alternative two markets from the stock, oil, gold, currency, and cryptocurrency markets.

^9^The two time-dummy variables divide the study period into the pre- and post-COVID-19 periods according to the date of the COVID-19 pandemic crisis occurring. BEKK is the abbreviation of the authors of Baba et al. (35) whereas GARCH is the abbreviation “Generalized AutoRegressive Conditional Heteroskedasticity.”

^10^Even though, this study uses a model with two time-dummy variables to explore the impact of the COVID-19 pandemic on the interaction issues. As shown in Table 2B, there are still some kinds of literature investigating the impact of the COVID-19 pandemic on the spillover or correlation issues by using RavenPack Coronavirus Media Coverage Index or Ravenpack Coronavirus Panic Index (25, 27–32, 34). In addition, Zaremba et al. (24) evaluated the impact of COVID-19 on the term spreads of bonds by using the change in the number of COVID-19 infections.

^11^The bivariate GARCH family models are composed of the mean equation and variance-covariance equation. The mean equation is expressed in the bivariate vector autoregressive (VAR) form. On the other hand, the variance-covariance equation is expressed as the BEKK-GARCH model of Baba et al. (35), the DCC-GARCH model of Engle (37), or the VAR-GARCH model of Ling and McAleer (8).

^12^Erdogan et al. (16) used the bivariate VAR model with the Granger causality test and the causality-in-variance test of Hafner and Herwartz (17) to, respectively, examine the return spillover and volatility spillover for a pair of assets.

^13^For example, Hong's (11) causality-in-mean and causality-in-variance tests can't determine whether the values of the return and volatility spillovers are positive or negative. Further, they can't find any result of correlation. Moreover, the TVP-VAR methodology can provide a time-varying spillover index expressed as a percentile value for the return and volatility spillovers. However, it can't give statistically significant results of return and volatility spillovers. Also, it can't determine whether the values of the return and volatility spillovers are positive or negative. Further, they can't find any result of correlation. Furthermore, the wavelet coherence approach can't give statistically significant results of the correlation. Further, they can't find any results of return and volatility spillovers.

^14^The BEKK type of a model is named after Baba et al. (35). Additionally, Su (39) adopted the suggestion of Moschini and Myers (40) to simplify the BEKK model and then he proposed a positive definite type of the bivariate BEKK-GARCH model in the diagonal representation to let the parameters estimate be parsimony. Hence, this diagonal bivariate variance–covariance specification owns two properties: first, the positive definite in the covariance matrix and second, the parsimony in the parameter estimation and thus the easiness in the parameter explanation. Please refer to Su (39) for more details.

^15^In this study, to achieve the risk diversification we use the minimum variance portfolios strategy to execute the optimal asset allocation whereas Esparcia et al. (43) uses a minimum VaR portfolios strategy to perform the optimal asset allocation.

^16^The top 10 most traded currencies in the world are involved in nearly 90% of trades. They are US dollar (USD), Euro (EUR), Japanese yen (JPY), Pound sterling (GBP), Australian dollar (AUD), Canadian dollar (CAD), Swiss franc (CHF), Chinese renminbi (CNY), Hong Kong dollar (HKD), New Zealand dollar (NZD).

^17^All cryptocurrencies other than Bitcoin are called altcoins, and they often use different protocols and encryption algorithms to overcome some of Bitcoin's limitations.

^18^On March 11, 2020, the Director General of the World Health Organization (WHO) defined the COVID-19 outbreak as a worldwide pandemic.

^19^The number in the bracket beside the title of paired market denotes the total number of pairs of assets in that paired market. For example, in the oil-cryptocurrency paired market, there are three pairs of assets such as wt-bi, wt-et, and wt-li. Hence, the total number of pairs of assets is 3. The symbol “wt” denotes the WTI crude oil in the oil market whereas the symbols “bi,” “et,” and “li” represent the Bitcoin, Ethereum, and Litecoin in the cryptocurrency market, respectively.

^20^Umar et al. (32) used Granger-causality test results to construct the network graphs of return spillover and volatility spillover. In addition, the summary results of the stock-oil, stock-gold, and oil-gold paired market are the results listed in the columns “stock-oil,” “stock-gold,” and “oil-gold,” respectively.

^21^Su (14) and Sui and Sun (15) found that there exists a return spillover from the currency market to the stock market whereas Su (19), Yang and Doong (12), Kumar (13), and Erdogan et al. (16) discovered that there exists a return spillover from the stock market to the currency market.

^22^Su (19) found that there exists a return spillover from the stock market to the oil market in the pre-SB period and Uzonwanne (18) found that there exists a return spillover from the stock market to the cryptocurrency market for the FTSE-bitcoin pair of data. Conversely, Yousaf and Hassan (9) discovered that there exists a return spillover from the oil market to the stock market during the US subprime crisis and the Chinese stock market crash.

^23^Yousaf et al. (22) used the VAR-DCC-GARCH model to analyze the return and volatility transmission of oil-gold and oil-Bitcoin pairs during the pre- and COVID-19 periods. They found that a return transmission is significant from bitcoin to the oil market (i.e., bitcoin → oil) during the COVID-19 period.

^24^Gomez-Gonzalez et al. (46) and Jebabli et al. (47) found that there exists a volatility spillover from the stock market to the oil market and Su (19) also found this result appears in the pre-SB period. In addition, Uzonwanne (18) found that there exists a volatility spillover from the stock market to the cryptocurrency market.

^25^Yousaf et al. (22) found that there exists a unidirectional volatility spillover from gold to oil (i.e., gold → oil) in the pre-COVID-19 period.

^26^Yousaf et al. (22) found that there is a unidirectional volatility spillover from bitcoin to oil (i.e., bitcoin → oil) in the pre-COVID-19 period.

^27^Gomez-Gonzalez et al. (46) and Jebabli et al. (47) found that there exists a volatility spillover from the stock market to the oil market and Su (19) also found this result appears in the pre-SB period. In addition, Yousaf et al. (22) found that there exists a volatility spillover from the gold market to the oil market in the pre-COVID-19 period.

^28^In addition, there exists four **cases** of slightly significant volatility spillovers for the pre-COVID-19 period but five for the post-COVID-19 period. Due to the limited space, we omit the discussion for the above slightly significant case.

^29^Su (19) found that there exists a significant correlation between the stock market and the oil market.

^30^In addition, there exists two **cases** of slightly significant correlation for the pre-COVID-19 period but four for the post-COVID-19 period. Due to the limited space, we omit the discussion for the above slightly significant case.

^31^The number in the bracket beside a specific type of interaction denotes the total number of paired markets that are significant for that specific type of interaction.

^32^Because the difference between the two total number of paired markets, that are significant, respectively, for the two periods, for the volatility spillover is greater than that for the return spillover and correlation.

^33^Owing to the following two reasons, the fund managers may construct a portfolio by the assets in the stock, oil, gold, currency, and cryptocurrency markets to diversify the risk according to the level of risk aversion of customers. The first reason is that oil (WTI), gold, two currencies, and three different cryptocurrencies have been traded in commodity market, currency market, and cryptocurrency market now. The second reason is that, as reported in section of Data and descriptive statistics, the assets in different market have not the nearly same trend of price. Moreover, they have the different attributes on the return and risk.

^34^The first hedged portfolio takes a long position on the first asset and a short position on the second asset. On the other hand, the second hedged portfolio takes a long position on the second asset and a short position on the first asset.

^35^We omit the dj-ch pair of assets in the comparison of the first group because the dj-ch pair of assets is suitable to construct the first hedged portfolio whereas the other two pairs of assets (dj-wt and dj-et) are suitable to establish the second hedged portfolio.

^36^From the scales of the vertical axis in Figures 7A–C, we find that the smallest value of “β_1_” appears at the wt-et during the pre- and post-COVID-19 periods. From Figures 8A–C, we also find, as compared with the other two pairs of assets, the values of “$HE_{1}^{h}$” for the wt-go are >0 for most of the time during the pre-COVID-19 period. Conversely, the values of $”HE_{1}^{h}$” for the wt-eu are >0 for most of the time during the post-COVID-19 period.

^37^From the scales of the vertical axis in Figures 7D,E, we find that the value of “β_1_” for the go-et is smaller than that for the go-eu at the pre- and post-COVID-19 periods.

^38^From the scales of the vertical axis in Figures 8D,E, we find that the value of “$HE_{1}^{h}$” for the go-eu is greater than that for the go-et at the pre- and post-COVID-19 periods.

^39^We can use the findings about the assets in the cryptocurrency market to help the fund manager make a decision in the issues of asset allocation and hedged strategy. Then, some policy implications for the investors and fund managers are proposed in the next paragraph. For example, in the issues of asset allocation, we can't involve the assets in the cryptocurrency market in a portfolio because as reported in the analysis of data and descriptive statistics they have the higher risk in each subperiod. Conversely, we can select the Ethereum as the hedge asset for gold or WTI in each subperiod because the Ethereum is the optimal hedge asset of gold or WTI during the pre-COVID-19 and post-COVID-19 periods from the perspective of hedge cost.
